# Clinical characteristics and outcomes of critically ill mechanically ventilated COVID-19 patients receiving interleukin-6 receptor antagonists and corticosteroid therapy: a preliminary report from a multinational registry

**DOI:** 10.1186/s40001-021-00591-x

**Published:** 2021-10-02

**Authors:** Marwa Amer, Ahmed M. Kamel, Mohammed Bawazeer, Khalid Maghrabi, Abid Butt, Talal Dahhan, Eiad Kseibi, Syed Moazzum Khurshid, Mohammed Abujazar, Razan Alghunaim, Muath Rabee, Maal Abualkhair, Ali Al-Janoubi, Abeer Turki AlFirm, Ognjen Gajic, Allan J. Walkey, Jarrod M. Mosier, Igor Borisovich Zabolotskikh, Oscar Y. Gavidia, Santiago Yus- Teruel, Michael A. Bernstein, Karen Boman, Vishakha K. Kumar, Vikas Bansal, Rahul Kashyap, the Society of Critical Care Medicine Discovery Viral Infection and Respiratory Illness Universal Study (VIRUS): COVID-19 Registry Investigator Group

**Affiliations:** 1 Pharmaceutical Care Division, King Faisal Specialist Hospital and Research Center, (MBC # 11), PO Box 3354, Riyadh, 11211 Saudi Arabia; 2 College of Medicine, Alfaisal University, Riyadh, Saudi Arabia; 3 Department of Critical Care Medicine, King Faisal Specialist Hospital and Research Center, Riyadh, Saudi Arabia; 4 Clinical Pharmacy Department, Faculty of Pharmacy, Cairo University, Giza, Egypt; 5 Biostatistics, Epidemiology & Scientific Computing Department, King Faisal Specialist Hospital and Research Center, Riyadh, Saudi Arabia; 6 Mayo Clinic, Rochester, USA; 7 Boston University School of Medicine, Boston, USA; 8 Banner University Medical Center-Tucson, Tucson, USA; 9 Kuban State Medical University with Affiliation Territorial Hospital #2, Krasnodar, Russia; 10 Clínica Medical Bogotá, Bogotá, Colombia; 11 Hospital Universitario La Paz, Madrid, Spain; 12 Stamford Health, Stamford, USA; 13 Society of Critical Care Medicine, Mount Prospect, USA

**Keywords:** Interleukin-6, Interleukin-6 receptor antagonist, Steroid, Critical care, Coronavirus disease 2019, Invasive mechanical ventilation

## Abstract

**Background:**

Interleukin-6 receptor antagonists (IL-6RAs) and steroids are emerging immunomodulatory therapies for severe and critical coronavirus disease (COVID-19). In this preliminary report, we aim to describe the epidemiology, clinical characteristics, and outcomes of adult critically ill COVID-19 patients, requiring invasive mechanical ventilation (iMV), and receiving IL-6RA and steroids therapy over the last 11 months.

**Materials and methods:**

International, multicenter, cohort study derived from Viral Infection and Respiratory Illness University Study registry and conducted through Discovery Network, Society of Critical Care Medicine. Data were collected between March 01, 2020, and January 10, 2021.

**Results:**

Of 860 patients who met eligibility criteria, 589 received steroids, 170 IL-6RAs, and 101 combinations. Patients who received IL-6RAs were younger (median age of 57.5 years vs. 61.1 and 61.8 years in the steroids and combination groups, respectively). The median C-reactive protein level was > 75 mg/L, indicating a hyperinflammatory phenotype. The median daily steroid dose was 7.5 mg dexamethasone or equivalent (interquartile range: 6–14 mg); 80.8% and 19.2% received low-dose and high-dose steroids, respectively. Of the patients who received IL-6RAs, the majority received one dose of tocilizumab and sarilumab (dose range of 600–800 mg for tocilizumab and 200–400 mg for sarilumab). Regarding the timing of administration, we observed that steroid and IL-6RA administration on day 0 of ICU admission was only 55.6% and 39.5%, respectively. By day 28, when compared with steroid use alone, IL-6RA use was associated with an adjusted incidence rate ratio (aIRR) of 1.12 (95% confidence interval [CI] 0.88, 1.4) for ventilator-free days, while combination therapy was associated with an aIRR of 0.83 (95% CI 0.6, 1.14). IL-6RA use was associated with an adjusted odds ratio (aOR) of 0.68 (95% CI 0.44, 1.07) for the 28-day mortality rate, while combination therapy was associated with an aOR of 1.07 (95% CI 0.67, 1.70). Liver dysfunction was higher in IL-6RA group (*p* = 0.04), while the bacteremia rate did not differ among groups.

**Conclusions:**

Discordance was observed between the registry utilization patterns (i.e., timing of steroids and IL-6RA administration) and new evidence from the recent randomized controlled trials and guideline recommendations. These data will help us to identify areas of improvement in prescribing patterns and enhance our understanding of IL-6RA safety with different steroid regimens. Further studies are needed to evaluate the drivers of hospital-level variation and their impact on clinical outcomes.

*Trial registration* ClinicalTrials.gov: NCT04486521. Registered on July 2020

**Supplementary Information:**

The online version contains supplementary material available at 10.1186/s40001-021-00591-x.

## Background

Severe and critical coronavirus disease 2019 (COVID-19) can manifest as respiratory failure with elevated inflammatory markers, resulting in exaggerated cytokine release, for which interleukin-6 receptor antagonists (IL-6RAs) are approved as treatment [1–3]. Interest in IL-6RAs and corticosteroids has increased recently due to their potential role as immunomodulators [4–7]. In view of the results from International Randomized, Embedded, Multi-factorial, Adaptive Platform trial for Community-Acquired Pneumonia (REMAP-CAP) and Randomized Evaluation of COVID-19 Therapy (RECOVERY), guidelines by National Institutes of Health and Infectious Disease Society of America conditionally suggest tocilizumab in combination with steroids (low-dose dexamethasone, 6 mg daily for 10 days) for intensive care unit (ICU) patients exhibiting rapid respiratory failure progression or high inflammatory markers [8–11]. On July 6, 2021, the World Health Organization (WHO) rapid evidence appraisal for COVID-19 therapies (REACT) working group developed a prospective meta-analysis of IL-6RAs in patients hospitalized for COVID-19 and showed that 28-day all-cause mortality rate was lower among patients who received IL-6RAs compared with those who received usual care or placebo [12]. The lower mortality rate was more marked among patients who received concomitant steroids and did not require invasive mechanical ventilation (iMV) at randomization. As such, WHO Living guideline recommends IL-6RAs (tocilizumab or sarilumab) in combination with steroids for patients with severe or critical COVID-19 [13]. Moreover, the Bayesian reanalysis of RECOVERY trial showed that hospitalized COVID-19 patients on non-invasive ventilation (NIV), and high-flow nasal cannula (HFNC) have a high probability of a clinically meaningful outcome benefit from tocilizumab [14]. Therefore, the immunomodulatory effect of IL6-RAs appears to be most beneficial in combination with steroids and when administered in the early phase of critical care trajectory.

A retrospective observational study from Viral Infection and Respiratory Illness Universal Study (VIRUS) registry during March–November 2020 identified a large hospital-level variation and geographic disparity in the use of repurposed medications for the management of COVID-19 [15]. Herein, we aimed to reflect on our experience over the past 11 months and to describe the epidemiology, clinical characteristics, and outcomes of critically ill adult COVID-19 patients requiring iMV and receiving IL-6RA and steroid therapy. This will help to identify areas of improvement in prescribing patterns to enhance adherence to the recent guideline recommendations, and subsequently the outcomes in this subgroup of patients.

## Materials and methods

### Study design and data source

Data for this study were derived from VIRUS registry, an international, multicenter, observational study conducted through Discovery Network, Society of Critical Care Medicine (SCCM), and included 168 hospitals across 16 countries. The study was approved by SCCM Scientific Review Committee, SCCM Discovery Steering Committee, and King Faisal Specialist Hospital and Research Center (KFSH&RC) Institutional Review Board (IRB) (IRB# 2201053, registered at ClinicalTrials.gov NCT04486521). In some participating hospitals, verbal consent was obtained from patients or surrogate decision-makers and documented in electronic medical records (EMRs); in other countries, consent was waived by local research ethics committees. Local investigators were responsible for obtaining local approval in line with applicable regulations. VIRUS is one of the largest registries that consecutively collects data on COVID-19 patients. Details of this database are described elsewhere [16, 17] (Additional file 1: Table S1). This study is reported following the Strengthening the Reporting of Observational Studies in Epidemiology (STROBE) and the Risk Of Bias In Nonrandomized Studies of Interventions (ROBINS-I) guidelines [18, 19].

### Study population

Adult patients (18–85 years old) were eligible if admitted to ICU from March 01, 2020 to January 10, 2021, required iMV, had positive polymerase chain reaction (PCR) SARS-CoV-2, and received IL-6RAs (tocilizumab or sarilumab), corticosteroids (dexamethasone, hydrocortisone, methylprednisolone, or prednisone), or combination. We excluded patients who were < 17 years, repeatedly admitted to ICU during the same hospital visit, on chronic systemic steroids at home or taking steroids for indications other than COVID-19, or died before receiving IL-6RAs or steroids. We stratified the population into three groups: IL-6RA, corticosteroid, and combination. Corticosteroid group was stratified further based on dexamethasone equivalent dose (mg) into high and low-dose. High-dose was defined as > 15 mg/day of dexamethasone or equivalent. This cut-off was chosen based on the data from prior literature [20, 21]. The patients were followed for clinical outcomes and adverse events (AEs) up to day 28.

### Data collection

The following variables were collected on days 0 (ICU admission), 1–3, 7, 14, 21, and 28 from EMRs according to standard operating procedure: demographics and comorbidities (including immunocompromised patients such as patients with solid tumors, hematological malignancy, metastatic cancer, history of solid organ or bone marrow transplant, and HIV), biomarkers and labs [ferritin, interleukin-6 (IL-6), d-dimer, fibrinogen, C-reactive protein (CRP), lactate dehydrogenase (LDH), and lymphocyte count], microbiology, concomitant medications, MV duration, discharge status, PaO_2_(mmHg)/FiO_2_ (arterial partial pressure of oxygen over fractional inspired oxygen concentration; PF ratio), and timing of drug initiation and dose. Data were collected online and stored on a secure data server using Mayo Clinic’s Research Electronic Data Capture application (REDCap). Data quality was assessed routinely to ensure completeness and accuracy. We reported the clinical outcomes for ventilator-free days (VFDs) from iMV at day 28, which was chosen as a patient-centered outcome and highly influenced by mortality [22]. VFDs were defined as the number of days between successful weaning of MV and day 28 after study enrollment. Patients who were on MV and died before day 28 were determined to have 0 VFDs. We also reported ICU and hospital mortality, 28-day mortality, hospital and ICU length of stay (LOS), median change in PF ratio, and AEs. Details on outcome definitions and variables collected are provided in Additional file 1: Table S2.

### Statistical analyses

Sample size was determined pragmatically, based on all available ICU patients in VIRUS database who met eligibility criteria. Statistical analyses were performed using R software, V3.6.3 (Vienna, Austria). Counts and percentages were used to represent categorical variables. Continuous data were summarized using means ± standard deviations (SDs) or medians [interquartile ranges (IQR)]. Chi-square test was used to compare distribution of categorical variables, and Kruskal–Wallis test and ANOVA were used to compare distributions of continuous non-normal and normal variables, respectively. For outcome analysis, we used three statistical models. In model 1, marginal structural model (MSM) was used to compare three regimens after adjusting for non-time and time-varying covariates and included the overall treatment strategy not solely restricted to the first 48 h [23]. Observations were weighted by inverse probability of treatment weight (IPTW). Nonparametric modeling using generalized boosted model (GBM) estimate IPTWs with automatic handling of missing data [24]. TWANG package in R was used for analysis. Covariate distributions were compared before and after applying propensity weights. Standardized mean difference (SMD) was used to assess the balance of pseudo-population using Kolmogorov–Smirnov stop rule. Average treatment effect (ATE) estimation method was used throughout analysis of entire sample. Covariates included in model were judged as likely to influence outcomes and have been identified in several studies: age; sex; ethnicity; asthma/chronic obstructive pulmonary disease (COPD); acute respiratory distress syndrome (ARDS) grade; history of diabetes, hypertension, coronary heart disease, or congestive heart failure; the lowest FiO_2_; therapeutic anticoagulation; hydroxychloroquine; azithromycin; and antivirals (including remdesivir); vasopressors; and paralytics (details available at Additional file 2). Analyses were performed using robust variance estimators, taking into account clustering within hospitals and patients. Quasi-Poisson generalized linear modeling was used to compare VFDs after weighting. Quasi-Poisson regression coefficients were exponentiated to obtain incidence rate ratio (IRR), which is the expected change in VF-days (ratio), compared to reference category. Quasibinomial generalized linear modeling was used to compare mortality between three groups. Weighted Kaplan–Maier estimator was used to compare hospital and ICU LOS. Patients who were still in ICU after a 28-day study period were censored at 28 days. Log-rank test was used to compare survival before and after weighting. Cause-specific Cox regression analysis was used to assess factors associated with mortality at 28 days. Discharge and mortality were included as competing risks. Linear mixed modeling was used to compare changes in PF ratio. Hypothesis testing was performed at 5% level of significance.

Model 2 included exploratory analyses of potential factors associated with variation in VFDs and mortality by adding Sequential Organ Failure Assessment (SOFA) score and highest FiO_2_ to the base model 1.

In model 3, we performed a sensitivity analysis to emulate target trial to reduce immortal time bias, which is restricted to the treatment received in the first 48 h. Patients eligible for target trial approach were those on iMV, received IL-6RAs or steroids within ICU days 0 or 1. Patients who died within first 48 h were excluded. Those who get treatment of interest after day 1 were classified by treatment exposure in first 48 h [similar to intention-to-treat analysis in randomized controlled trial (RCT)] [25]. The outcomes of interest in target trial approach were VFDs and 28-day mortality. Post hoc analyses were conducted for clinical outcomes stratified by age (> 60 vs. ≤ 60) and to examine changes in biomarkers over time using linear mixed models.

## Results

**Fig. 1:**
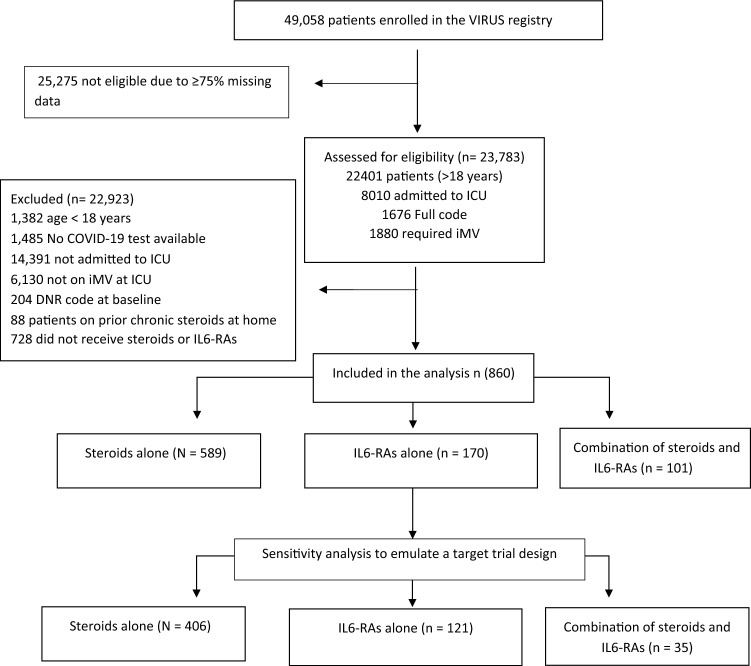
Flowchart for data extraction from VIRUS database. *DNR* do-not-resuscitate order, *ICU* intensive care unit, *iMV* invasive mechanical ventilation, *IL6-RAs* interleukin-6 receptor antagonists, *VIRUS* Viral Infection and Respiratory Illness University Study registry

A total of 23,783 patients were screened; 860 met eligibility criteria and were classified as follows: 170 received IL-6RA, 589 received steroids, and 101 received both therapies. In sensitivity analysis to emulate target trial, 562 patients fulfilled inclusion criteria: 406 received steroids (72.2%), 121 received IL6-RAs (21.5%), and 35 received both (6.23%) (Fig. 1).

### Patient characteristics

In the unadjusted analysis presented in Additional file 1: Table S3, baseline characteristics were not balanced. Patients who received IL-6RAs were younger (median age 57.5 years vs. 61.1 and 61.8 years in the steroid and combination groups, respectively; *p* = 0.009), and chronic pulmonary diseases, excluding asthma (COPD, bronchiectasis, and interstitial lung disease) were more prevalent in combination group (16 [15.8%] vs. 7 [4.12%] and 59 [10%] in IL-6RA and steroid groups, respectively; *p* = 0.005). The ARDS was prevalent in 35.5% of steroid-treated patients, 14.7% in IL-6RAs, and 36.6% in combination therapy; most of these patients had severe ARDS grades (PF ratio < 100). Patients in IL-6RAs and combination groups presented more frequently with fever, nasal congestion, and rhinorrhea than those in steroid group, while myalgia or fatigue was most frequently reported in steroid group. The incidence of dyspnea and shortness of breath was similar among the three groups. After adjusting for baseline covariates, SMD was ~ 0.1 for the majority of the covariates, indicating that the three groups were well-balanced (Table 1).

**Table 1:** Baseline characteristics after adjusting for baseline covariates

Variables	Steroids	IL6-RAs	Both	*P*	SMD
*N*^a^	811.6	596.8	698.9		
Male	528 (65.1)	430 (72.1)	433 (62)	0.25	0.145
Age [mean (SD)]	60.9 (14.2)	60.6 (13.8)	61.5 (12.8)	0.90	0.042
Weight at admission [mean (SD)]	91.3 (27.9)	92.7 (26.3)	94.5 (29.9)	0.62	0.076
Ethnic group				0.93	0.113
Hispanic	163 (20.1)	128 (21.5)	147 (21.3)		
Non-hispanic	385 (47.4)	285 (47.8)	325 (47)		
CVD	176 (21.6)	75 (12.6)	161 (23)	0.11	0.183
Asthma/COPD	143 (17.6)	84 (14.1)	147 (21)	0.33	0.121
Hydroxychloroquine	164 (20.2)	181 (30.2)	180 (26)	0.15	0.141
Azithromycin	300 (37)	279 (46.7)	294 (42.1)	0.32	0.122
Antiviral	145 (18)	78 (13.1)	141 (20.2)	0.42	0.136
ARDS grade				0.79	0.148
Mild (*P*:*F* 200–300)	22 (2.8)	17 (3)	23 (3.3)		
Moderate (*P*:*F* 100–199)	83 (10.7)	38 (6.5)	41 (6)		
Severe (*P*:*F* < 100)	132 (16.9)	79 (13.8)	113 (16.2)		
Any anticoagulant	693 (85.3)	504 (84.4)	560 (80.1)	0.46	0.093
Therapeutic anticoagulation	179 (22.1)	87 (14.6)	162 (23.1)	0.15	0.147
FiO_2_ lowest [mean (SD)]	0.56 (0.25)	0.52 (0.24)	0.57 (0.24)	0.43	0.127
Neuromuscular blocker	659 (82)	520 (87)	625 (89.4)	0.15	0.16
Vasopressors	638 (78.6)	506 (84.8)	612 (87.5)	0.14	0.16

Data presented as *n* (%) unless otherwise specified

*ARDS* acute respiratory distress syndrome, *CVD* cardiovascular diseases, *COPD* chronic obstructive pulmonary disease, *IL6-RAs* interleukin-6 receptor antagonists, *SMD* standardized mean difference, *SD* standard deviation, *P:F* ratio of arterial oxygen partial pressure (PaO_2_ in mmHg) to fractional inspired oxygen

^a^The total number is slightly different in the post-IPW pseudo-data set as a result of the weighting. Each individual gets own weight that is used for further analysis. Multiplication by these weights can usually results in decimals

Table 2 illustrates the biomarker and laboratory levels at ICU day 0 after adjusting for covariates. The CRP level (assessed in 413) was > 75 mg/L across all groups, while IL-6 concentrations (assessed in 24) were highest in the combination group. The FiO_2_ was the highest in the combination group during the first 24 h. The mean LDH concentration (µkat/L) was 12.66 [SD 8.84] in steroid, 9.65 [SD 5.03] in IL-6RAs, and 9.46 [SD 5.32] in combination groups (*p* = 0.001).

**Table 2:** Laboratory parameters and biomarkers at ICU day 0 after adjusting of covariates

Variables	Steroids	IL6-RAs	Both	*P*	SMD
*N*^a^	804.07	572.13	693.34		
Blood glucose (highest)	10.47 (5.54)	9.88 (5.13)	10.87 (5.55)	0.55	0.122
FiO_2_% (0.21 to 1.0) (lowest)	0.56 (0.25)	0.52 (0.24)	0.57 (0.24)	0.34	0.145
FiO_2_% (0.21 to 1.0) (highest)	0.79 (0.25)	0.72 (0.26)	0.84 (0.22)	**0.01**	0.33
FiO_2_ (0.21 to 1.0) at the time of blood gas	0.76 (0.56)	0.62 (0.28)	0.73 (0.26)	**0.02**	0.259
Arterial PaO_2_ at time of FiO_2_	88.94 (51.19)	84.16 (30.70)	91.54 (47.87)	0.56	0.116
Alanine aminotransferase (ALT/SGPT) (highest)	0.82 (0.76)	0.96 (1.25)	0.92 (1.14)	0.66	0.086
Aspartate aminotransferase (AST/ SGOT) (highest)	1.18 (1.25)	1.29 (1.34)	1.34 (1.74)	0.71	0.074
Alkaline phosphatase (highest)	1.49 (0.90)	1.44 (0.77)	1.69 (1.06)	0.43	0.177
Total bilirubin (highest)	13.85 (11.45)	10.81 (8.43)	13.04 (11.99)	0.033	0.195
C-reactive protein (CRP) (highest)	144.59 (111.78)	122.95 (108.34)	142.54 (100.90)	0.38	0.134
Ferritin (highest)	1264.92 (1272.46)	1918.15 (4028.79)	1379.02 (1710.94)	0.40	0.156
Lactate dehydrogenase (LDH) (highest)	12.66 (8.84)	9.65 (5.03)	9.46 (5.32)	**0.001**	0.298
Leukocyte count (lowest)	10.69 (6.39)	8.56 (5.52)	10.10 (4.54)	**0.02**	0.256
Leukocyte count (highest)	11.13 (6.65)	9.22 (5.28)	10.78 (5.79)	**0.01**	0.219
Lymphocyte count (lowest)	15.79 (19.99)	13.32 (10.84)	13.89 (12.14)	0.28	0.106
d-Dimer	1746.03 (2726.93)	1193.26 (2758.81)	1064.68 (3171.54)	0.20	0.158
Procalcitonin (highest)	2.20 (5.19)	0.88 (1.98)	1.11 (3.17)	**0.02**	0.225
Fibrinogen level (highest)	6.19 (2.09)	6.17 (1.56)	6.40 (2.01)	0.88	0.078
Interleukin 6 (IL-6) (highest)	123.27 (162.97)	202.84 (196.81)	283.06 (228.51)	0.31	0.541
Highest lactate	2.44 (2.05)	1.86 (0.78)	2.22 (1.34)	**0.005**	0.273

Data presented as mean (SD). Units presented as SI units for the following: PaO_2_ in mmHg; ALT/SGPT, AST/SGOT, alkaline phosphatase, and lactate dehydrogenase (µkat/L); bilirubin (µmol/L); ferritin (mcg/L); leukocytes (×10^9^/L); d-dimer mg/L; procalcitonin (mcg/L); fibrinogen (g/L); interleukin 6 (il-6) (pg/mL); lactate (mmol/L); platelet (×10^9^/L)

The following presented as conventional unit: lymphocyte (% of white blood cells); C-reactive protein (mg/L)

FiO_2_: fractional inspired oxygen; IL6-RAs: interleukin-6 receptor antagonists; PaO_2_: arterial oxygen partial pressure

^a^The total number is slightly different in the post-IPW pseudo-dataset as a result of the weighting

Bold values indicate a significant value at 5% level of significance

Table 3 summarizes the utilization pattern of steroids and IL6-RAs in relation to ICU admission. The median daily steroid dose was 7.5 mg dexamethasone equivalent (IQR 6–14 mg). Of these, 80.8% received low-dose steroids, and 19.2% received high-dose steroids. Of the patients who received IL-6RAs, the majority (84.5%) received one dose of tocilizumab, while second and third doses were administered to 13.1% and 2.3% of patients, respectively. Similarly, most patients (84.7%) in sarilumab group received a single dose. The dosage range of tocilizumab was 600–800 mg, and sarilumab was 200–400 mg. Regarding the timing of the IL-6RA administration, 39.5% were received on day 0 of ICU admission, and ~ 30% started on days 1 and 2 (20.3% and 11.1%, respectively).

**Table 3:** Utilization pattern of steroids and IL6-RAs in relation to ICU admission

Variables	Value
Average daily dose (mg dexamethasone equivalent)^a^	7.50 [6, 14.1]
Low steroid dose	496 (80.8)
High steroid dose^b^	118 (19.2)
Day of steroid start^c^	
0	384 (55.6)
1	102 (14.8)
2	37 (5.4)
3	22 (3.2)
4	24 (3.5)
After day 5	118 (17)
Tocilizumab	213 (78.3)
Tocilizumab number of doses^d^	
1	180 (84.5)
2	28 (13.1)
3	5 (2.3)
Sarilumab	59 (21.7)
Sarilumab number of doses^d^	
1	50 (84.7)
2	9 (15.3)
Day of IL6-RAs start	
0	129 (39.5)
1	55 (20.3)
2	30 (11.1)
3	21 (7.8)
4	4 (1.5)
After day 5	32 (11.8)

Data presented as *n* (%) unless otherwise specified

*IL6-RAs* interleukin-6 receptor antagonists

^a^The dose was available to 614 patients. The average daily dose of steroids was calculated by averaging the daily doses received by the patients during the ICU stay

^b^High dose was defined as > 15 mg/day of dexamethasone or dexamethasone equivalent

^c^The proportion was calculated from the number of patients who received the steroids (*n* = 693)

^d^The proportion was calculated from the number of patients who received IL6-R antagonists intravenous formulation (*n* = 272)

Additional comorbidities, ventilator and radiological characteristics, and ICU supports are included in Additional file 1: Tables S4–S9. IL-6RAs and the combination arm had more multifocal and bilateral interstitial patterns on chest radiography on day 1 of the ICU stay. Matched patients received comparable amounts of sedatives, paralytics, and anticoagulation.

### Outcome data

The results for outcomes before adjusting for covariates are displayed in Additional file 1: Table S10. Table 4 summarizes the main clinical outcomes data. When compared to steroid alone by day 28, the use of IL-6RAs was associated with adjusted aIRR of 1.12 (95% CI 0.88, 1.4) for VFDs, while combination therapy was associated with aIRR of 0.83 (95% CI 0.6, 1.14) (model 1). Exploratory analysis findings (model 2) were comparable to primary analysis. Sensitivity analysis of target trial design (model 3) showed consistent results when compared to steroids alone (aIRR [95% CI]): IL-6RAs (1.13 [0.87, 1.45]), combination (0.64 [0.34, 1.23]). Linear regression analysis was performed stratified by steroid dose (Additional file 1: Table S11). Compared to IL-6RAs, low-dose steroid was associated with VFDs *β* value of 0.62 (95% CI − 1.54, 2.78) and high-dose steroid was associated with VFD *β* value of − 1.19 (95% CI − 3.85, 1.47). Factors associated with a higher likelihood of VFDs were non-use of paralytics, therapeutic anticoagulation use, and low FiO_2_.

**Table 4:** Clinical outcomes data

Association between treatment modality and ventilator-free days	aIRR (95% CI)
Steroids	Reference
IL6-RAs	
VFD (model 1)	1.12 (0.88, 1.40)
VFD (model 2 with highest FiO_2_ added)	1.13 (0.90, 1.42)
VFD (model 2 with SOFA added)	1.12 (0.89, 1.40)
Target trial design (model 3)	1.13 (0.87, 1.45)
Combination therapy	
VFD (model 1)	0.83 (0.60, 1.14)
VFD (model 2 with highest FiO_2_ added)	0.88 (0.64, 1.20)
VFD (model 2 with SOFA added)	0.77 (0.56, 1.06)
Target trial design (model 3)	0.64 (0.34, 1.23)

**Association between treatment modality and 28-day mortality**	**aOR (95% CI)**
Steroids *N* = 583	Reference
IL6-RAs	
28-day mortality (model 1) *N* = 168	0.68 (0.44; 1.07)
Hospital morality (model 1) *N* = 170	0.68 (0.43, 1.09)
28-day mortality (model 2) *N* = 170	0.7 (0.45, 1.08)
Target trial design (model 3) *N* = 121	0.6 (0.35, 1.03)
Combination therapy	
28-day mortality (model 1) *N* = 104	1.07 (0.67, 1.70)
Hospital morality (model 1) *N* = 101	1.23 (0.72, 2.11)
28-day mortality (model 2) *N* = 101	1.21 (0.76, 1.93)
Target trial design (model 3) *N* = 35	1.96 (0.90, 4.28)

Missing data outcomes in sensitivity analysis (model 3): 4 patients had 28-day mortality status missing, and 15 patients had duration of iMV missing

Adjusted incident rate ratio (aIRR) was used for VFD (> 1 is favorable for intervention), Adjusted odds ratio (aOR) was used for 28-day mortality (< 1 is favorable for intervention)

*aIRR* adjusted incident rate ratio, *aOR* adjusted odds ratio, *CI* confidence interval, *FiO*_*2*_ fractional inspired oxygen concentration, *IL6-RAs* interleukin-6 receptor antagonists, *SOFA* Sequential Organ Failure Assessment, *VFDs* ventilation-free days

When compared to steroid alone by day 28, IL-6RAs was associated with adjusted odds ratio (aOR) of 0.68 (95% CI 0.44, 1.07) for the mortality rate, while combination was associated with aOR of 1.07 (95% CI 0.67, 1.70) (model 1). Similarly, exploratory analyses (model 2), sensitivity analysis findings of target trial approach (model 3), and hospital mortality were consistent with primary analysis when compared to steroids (hospital mortality aOR [95% CI]: IL6-RA (0.68, [0.43, 1.09]), combination (1.23, [0.72, 2.11]). Regarding the AEs, liver dysfunction was higher in IL-6RAs (*p* = 0.04), while bacteremia rate did not differ among groups (Table 5). Detailed data of other outcomes are available in Additional file 1: Tables S12–S16, Figures S1–S5, and Additional file 3. Binary logistic regression was used to assess 28-day mortality stratified by steroid dose and showed no significant association between the use of steroids (low- or high-dose) and mortality**.** Post hoc analysis for the outcomes (VFDs and mortality) stratified by age (> 60 vs. ≤ 60) showed that younger patients perhaps had more favorable outcomes with IL-6RA compared with the older population. We examined the changes in biomarkers (LDH, CRP, and ferritin) over time using linear mixed models for repeated measurements. On day 28, both IL-6RAs [coefficients *β* = − 0.98] and combination groups [coefficients *β* = − 3.39] had a clinically meaningful reduction in CRP than the steroids-only group (reference group). Similarly, IL-6RAs [coefficients *β* = − 51.65] and combination groups [coefficients *β* = − 32.19] had a clinically meaningful reduction in ferritin than the steroid. No reduction was observed in LDH levels in IL-6RAs or combination groups.

**Table 5:** Documented complication during hospitalization after adjusting for baseline covariates

Variables	Steroids	IL6-RAs	Both	*p*
*N*^a^	804.1	582.4	689.6	
None	35 (4.3)	40 (6.8)	16 (2.3)	0.30
Acute cardiac injury	63 (7.9)	56 (9.6)	33 (4.8)	0.32
Acute kidney injury	292 (36.4)	280 (48.1)	277 (40.2)	0.15
Cardiac arrest	155 (19.3)	84 (14.4)	114 (16.5)	0.52
Cardiac arrhythmia new onset	101 (12.6)	67 (11.4)	66 (9.6)	0.72
Coagulation disorder disseminated intravascular coagulation	34 (4.3)	59 (10.0)	50 (7.3)	0.23
Deep vein thrombosis	51 (6.3)	59 (10.1)	68 ( 9.9)	0.34
Empyema	3 (0.3)	5 (0.9)	5 (0.7)	0.70
High troponin level	97 (12.1)	57 (9.8)	134 (19.4)	**0.04**
Liver dysfunction/acute liver failure	118 (14.6)	139 (23.9)	82 (11.9)	**0.04**
Lung abscesses	3 (0.3)	0 (0)	5 (0.7)	0.45
Myocarditis	8 (1.0)	7 (1.3)	0 (0)	0.28
Pneumothorax	54 (6.8)	35 (6.0)	30 (4.4)	0.64
Causative microbiology and rate of secondary infections				
Respiratory viral panel (RVP) PCR positive^b^	1 (0.2)	2 (1.2)	0 (0)	0.72
Bacterial pneumonia	192 (23.9)	173 (29.7)	197 (28.5)	0.46
Cytomegalovirus PCR positive	2 (0.3)	0 (0)	1 (0.9)	0.40
Legionella urine Ag positive	0 (0)	1 (0.6)	0 (0)	0.32
Blood cultures positive^c^	93 (21.9)	27 (17.2)	17 (19.3)	0.44
Hospital day(s) positive blood cultures performed, median [IQR]	7 [0.25; 14]	14 [1; 22]	10 [0.75; 13]	0.14
Sputum culture positive^d^	167 (50.6)	65 (53.3)	34 (45.9)	0.61
Hospital day(s) positive sputum culture performed, median [IQR]	4 [1; 11]	5 [1.5; 9]	5 [1; 13.5]	0.87
Urine culture positive^e^	74 (29.2)	23 (20.2)	11 (19.6)	0.10

Data presented as *n* (%) unless otherwise specified

*IL6-RAs* interleukin-6 receptor antagonists

^a^Each individual gets its own weight that is used for further analysis. Multiplication by these weights can usually results in decimals

^b^Performed in 127 individuals in steroids group, 85 in IL6-RAs group, 33 in combination group

^c^Performed in 424 individuals in steroids group, 157 in IL6-RAs group, 88 in combination group

^d^Performed in 331 individuals in steroids group, 121 in IL6-RAs group, 74 in combination group

^e^Performed in 254 individuals in steroids group, 114 in IL6-RAs group, 56 in combination group

Bold values indicate a significant value at 5% level of significance

## Discussion

The IL-6RAs were investigated in multiple RCTs, and some found lower duration of ICU and hospital stay, lower MV and death composite rates, and an increased number of organ support-free days in COVID-19 patients requiring respiratory support [26]. Herein, we reported characteristics and outcomes of adult ICU COVID-19 patients, who required iMV, and received IL-6RAs or steroids within a large, multinational registry over the past 11 months. We observed that the majority of our study cohort had a baseline CRP level of > 75 mg/L, indicating that our study population was skewed toward a hyperinflammatory phenotype and had higher baseline levels of critical illness, thereby supporting the mechanism of action of immunomodulators. Moreover, the number of tocilizumab and sarilumab doses that were administered in our study were comparable to those reported in prior RCTs, where the majority of the population received one dose of either drug.

Notably, our study included only patients on iMV (i.e., more severe end of the critical care trajectory), while recent RCTs included patients with varying degrees of respiratory support (iMV at baseline constitute 29% in REMAP-CAP, 14% in RECOVERY, 16% in TOCIBRAS, and 37% in COVACTA) [26]. We observed that IL-6RAs were associated with improvement in VFDs and 28-day mortality. We hypothesized possible reasons to explain these findings. First, monoclonal antibodies reside almost exclusively in the blood plasma and extracellular fluid due to their large size and hydrophilicity. Moreover, IL-6RAs have a long half-life (e.g., half-life of tocilizumab is roughly 13 days), resulting in a longer and persistent effect [5, 27]. Importantly, the incidence of secondary bacterial pneumonia was numerically higher in combination than IL-6RAs monotherapy group, possibly due to the additive immunosuppressant effect with combination therapy. These findings may indirectly relate to worse ICU mortality and prolonged ventilation days in combination group compared with IL-6RAs monotherapy. Likewise, recent studies have demonstrated the benefit of IL-6RAs in terms of the reduction in biomarkers, specifically CRP and ferritin levels [27]. Our study showed a clinically meaningful reduction in CRP and ferritin levels with IL-6RA use, perhaps another explanation for the observed favorable outcomes with this group. Lastly, there are potential other factors that could influence the observed outcomes over time. Changes in the severity of critical care patients, health system operational strain, the emergence of new COVID-19 variants, racial and ethnic differences in populations studied, and differences in the study conduction time, among others.

It is worth mentioning that the prescribing pattern and timing of administration observed in data registry for steroids and IL-6RA utilization were inconsistent with the best practice recommendation to optimize the effectiveness of immunomodulators. For example, we observed that only 55.6% of steroid administration occurred on day 0 of ICU admission. Ideally, this percentage should be closer to 100% in patients with iMV who are admitted to ICU, particularly since the announcement of RECOVERY steroids domain, indicating that the equipoise for withholding corticosteroids is no longer justifiable given the convincing evidence in favor of steroid use which now become standard care for ICU COVID-19 patients requiring MV [7]. One can argue that a portion of our data were collected before June 2020, when steroids use was limited due to the concerns of delayed viral clearance as shown in Middle East respiratory syndrome coronavirus [28]. Moreover, we also observed that IL-6RA administration was 39.48% on day 0 of ICU admission, 20.3% on day 1, and 11% on day 2, respectively. Ideally, this percentage in ICU patients with iMV should also be higher at baseline given the benefit of early administration of IL-6RAs, as observed in previous RCTs. In the REMAP-CAP, more than 75% of the participants received IL-6RAs within 3 days of hospital admission (i.e., early in their critical care trajectory) [8]. In WHO meta-analysis, a lower 28-day mortality rate was more marked among patients who received supplemental oxygen, NIV, and HFNC at randomization [12]. Similarly, the Bayesian reanalysis of RECOVERY trial showed that probabilities for a clinically significant mortality reduction (absolute risk difference > 3%) were 77%, 96%, and 56% in patients on simple oxygen, NIV, and iMV, respectively [14]. Taken together, the immunomodulatory effect of IL6-RAs and steroid appears to be most beneficial shortly following clinical deterioration at systemic hyper-inflammation onset and ideally before intubation, as their effect appears to be less pronounced if administered late for subsets of patients with iMV [29].

Additionally, we observed different types and doses of steroids, including high-dose steroids (19.2% of our study cohort). The REMAP-CAP included fixed duration steroids and shock-dependent hydrocortisone while RECOVERY used low-dose dexamethasone 6 mg IV daily. The Italian National Institute for Infectious Diseases recommends 5 days of methylprednisolone 1 mg/kg or dexamethasone 20 mg daily (defined as high-dose steroids in our study) [30]. This regimen is higher than the fixed dexamethasone dose used in RECOVERY and described in non-COVID ARDS literature [20, 21]. Prior studies suggested that moderate- to high-dose steroid regimens resulted in a greater reduction in mortality, organ dysfunction, MV requirement, and no increase in medical or infectious complications compared to low-dose regimens in ICU COVID-19 patients [31, 32]. In COVID STEROID 2 RCT (preprint), the use of 12 mg dexamethasone was compared to 6 mg dexamethasone in severe COVID-19 and there was no significant difference observed in the number of days alive without life support at day 28, or 28-day mortality. However, the 95% CI suggests that the results are most compatible with a benefit from higher dexamethasone dose (adjusted mean difference 1.3 days alive without life support [95% CI 0, 2.6], and 14% reduction in mortality [95% CI 0.68, 1.08]). An upcoming Bayesian analysis of COVID STEROID 2 RCT data will provide a more clinically meaningful interpretation to aid in the decision-making process [33]. Whether the beneficial immunomodulatory effects of IL-6R blockade could be achieved more easily and with less cost by using a different steroid regimen (higher dose) and its safety is unclear and need to be investigated in an adequately powered RCT [26].

Strengths of this study lie in its multinational nature. We used data from the largest COVID-19 registry and represented a spectrum of intensive care with racially and ethnically diverse cohorts. To our knowledge, we included relatively large numbers of patients on iMV at baseline, which was a limited subset of the population included in the previous trials. Additionally, we included laboratory-confirmed COVID-19 PCR tests, thereby minimizing selection or surveillance bias at each center. Lastly, prior observational studies could be biased by immortal time and indication bias [25, 34]. We used a novel statistical analysis to overcome these limitations and application of target trial design, which is likely a better approach to reduce immortal time bias and best resembling clinical practice. Although COVID-19 treatments were not used uniformly before matching and patients did not receive up-to-date standards of care, especially in early pandemic phases, we utilized rigorous methods to match the three groups concerning anticoagulation use, hydroxychloroquine, antivirals, and azithromycin. Importantly, this study highlights the risk of liver injury with IL-6 inhibition. Clinicians should be aware of these effects and weigh treatment risks and benefits accordingly. Whether this risk is intensified with repeated IL-6RA administration or related to patients’ critical presentation warrants investigation in future research.

We acknowledge some limitations. First, due to the observational nature of this study, the results for clinical outcome data are exploratory and possibly inconclusive due to insufficient power and frequentist statistical framework. Second, a fair number of patients were excluded due to missing outcomes and incomplete data for time-dependent confounders to be used in MSM. We may have missed patients who would have met the eligibility criteria, but were not included because of those reasons (possible elimination for non-random missingness). Although we used the best available methods to compare well-balanced groups, controlling for confounders in observational study may remain incomplete despite all efforts. Notably, some imbalances in potential confounders (e.g., sex, cardiovascular disease, respiratory diseases, and treatments offered) were observed after weighting (SMD > 0.1). Such potential residual confounding may have a role in the observed outcome findings. Moreover, there was a large proportion of incomplete data for SOFA and APACHE II scores due to the heavy burden of workload experienced by participating clinicians during the pandemic. Furthermore, our study included a relatively small sample size to examine changes in biomarkers (LDH, CRP, and ferritin) with immunomodulators and a lack of serial data to evaluate the association between the trajectory of other biomarkers and outcome (e.g., the potential role of IL-6 level in predicting IL-6RA responses). Steroid duration was also highly variable with inconsistent data entry, making it impossible to minimize its effect fully. Furthermore, this preliminary report is mainly focused on the first COVID-19 wave (March 01, 2020, to January 10, 2021) and before COVID-19 vaccines became widely available. We plan to include the COVID-19 vaccination status in subsequent reports of the data registry. Finally, our follow-up was limited to 28 days. Considering the risk of secondary infections and the apparent long half-life of IL-6RAs, it is logical to consider the need for long-term infection risk follow-up. Therefore, our data likely did not capture the true incidence of secondary infections attributed to IL-6RA or steroids, and a longer follow-up is needed to help characterize long-term sequelae, especially mucormycosis, aspergillosis, pneumocystis pneumonia, and multi-drug resistant organisms. Therefore, results should be interpreted within the limitations of the retrospective registry studies.

Through this preliminary report, we identified areas of improvement in prescribing patterns for steroids and IL-6RA utilization over the past 11 months, particularly in the timing of administration in patients requiring iMV. As the evidence has now become clearer, we expect that subsequent iterations of the registry data for steroid and IL-6RA utilization will be more consistent with the recent data coinciding with the accrual of scientific evidence.

## Conclusions

This study reports the features of critically ill mechanically ventilated COVID-19 patients receiving IL-6RA and steroid therapy. We observed that the prescribing pattern in the data registry over the past 11 months for these agents, particularly in the timing of administration, was inconsistent with the best practice recommendation set forth to optimize the effectiveness of immunomodulators, which may have resulted in the outcome findings observed here. The lessons learned from this study may help to identify areas of improvement in prescribing patterns, improve the decision-making process, and enhance our understanding of IL-6RA safety with different steroid regimens. Further studies are needed to evaluate the drivers of hospital variation and their impact on clinical outcomes.

## Supplementary Information

**Additional file 1.** Additional data and statistical analyses.

**Additional file 2.** Details for marginal structural model (MSM).

**Additional file 3.** Labs and biomarkers data on day 7, 14, 21.

**Additional file 4.** Collaborative authorship list.

## Data Availability

The data used to support the findings of this study are available from the corresponding author upon request.

## Abbreviations

ARDS: Acute respiratory distress syndrome
APACHE II: Acute Physiology and Chronic Health Evaluation II
ICU: Intensive care unit
IRB: Institutional Review Board
LOS: Length of stay
iMV: Invasive mechanical ventilation
SOFA: Sequential Organ Failure Assessment
COVID-19: Coronavirus disease 2019
IL-6RA: Interleukin-6 receptor antagonists
REMAP-CAP: Randomized, Embedded, Multi-factorial, Adaptive Platform Trial for Community-Acquired Pneumonia
RECOVERY: Randomized Evaluation of COVID-19 Therapy
NIV: Non-invasive ventilation
HFNC: High-flow nasal cannula
WHO: World Health Organization
MSM: Marginal structural model
SMD: Standardized mean difference
