# Supplementary material for: Clinical characteristics and outcomes of critically ill mechanically ventilated COVID-19 patients receiving interleukin-6 receptor antagonists and corticosteroid therapy: a preliminary report from a multinational registry
Source: Eur J Med Res. 2021 Oct 2;26:117. doi: 10.1186/s40001-021-00591-x (PMC8487342; doi:10.1186/s40001-021-00591-x)
Supplement: Supplementary file 1 — Additional file 1. Additional data and statistical analyses. [file 40001_2021_591_MOESM1_ESM.docx]

**Additional file 1. Additional data and statistical analyses**

| **Content** | **Page** |
| --- | --- |
| **Table S1:** Included hospitals | 2 |
| **Table S2:** Definitions of selected variables and clinical outcomes | 3 |
| **Table S3:** Distribution of baseline covariates across groups before matching | 4 |
| **Table S4:** Other baseline characteristics of the total cohort | 5-8 |
| **Table S5:**. Ventilator and radiology characteristics of the included patients at days 0 and 1 of ICU | 9-10 |
| **Table S6:** SOFA and APCHE-II scores at baseline | 11-12 |
| **Table S7:** ICU support and interventions at days 0 of ICU admission after IPTW and matching | 13 |
| **Table S8:** Distribution of Anakinra and Remdesivir doses | 14 |
| **Table S9:** When was the patient admitted to ICU with respect to hospital admission day. | 15 |
| **Table S10:**  Clinical outcomes before adjusting for non-time and time-varying covariates | 16 |
| **Table S11:** Linear regression analysis for ventilation-free days after adjusting for covariates stratified by steroid dose | 17 |
| **Table S12:** Association between medication use and mortality after adjustment stratified by steroid dose | 18 |
| **Table S13:** Other documented complication during hospitalization | 19 |
| **Table S14:** Post-hoc analysis for changes in selected biomarkers values using linear mixed modelling up to 28 days | 20 |
| **Table S15:** Linear mixed modelling of PF ratio | 21 |
| **Table S16:** Post-hoc analysis for the ventilator-free days outcome stratified by time of administration | 22 |
| **Figure S1:** Adjusted survival curves for competing risk models for (A) mortality and (B) discharge | 23 |
| **Figure S2:**Unweighted and weighted Kaplan–Maier estimates for association between treatment and ICU LOS | 24 |
| **Figure S3:**Unweighted and weighted Kaplan–Maier estimates for association between treatment hospital LOS | 24 |
| **Figure S4:** Estimated marginal trend for the change in PF ratio across groups. | 25 |
| **Figure S5:** Post-hoc analysis for the clinical outcomes ( ventilator-free days and mortality) stratified by age (> 60 vs. ≤ 60) | 26-27 |

**Table S1Included Hospitals**

| **Country** | **N** |
| --- | --- |
| 1. United States | 60 |
| 1. Japan | 4 |
| 1. Saudi Arabia | 4 |
| 1. India | 2 |
| 1. Belgium | 2 |
| 1. Colombia | 1 |
| 1. Mexico | 1 |
| 1. Bosnia and Herzegovina | 1 |
| 1. Croatia | 1 |
| 1. Egypt | 1 |
| 1. Hungary | 1 |
| 1. Hondurus | 1 |
| 1. Russia | 1 |
| 1. Serbia | 2 |
| 1. Spain | 1 |
| 1. Pakistan | 1 |
| **Total** | 84 |

These are the included hospital per country in the VIRUS registry

**Table S2:** **Definitions of Selected Variables and Clinical Outcomes**

| Severity of illness was evaluated using Sequential Organ Failure Assessment (SOFA) and Acute Physiology and Chronic Health Evaluation (APACHEII) scores. | The worst value for baseline laboratory tests was chosen. These data were collected within the first 24 h after admission to the ICU. If that data were not available, then data closest to admission were recorded. |
| --- | --- |
| Duration of mechanical ventilation | Recorded as the number of calendar days from intubation to extubation or until ICU discharge, or death whichever occurs first. The median ventilator-free days was calculated as calendar days with no ventilator support to day 28. Participants who die before day 28 are assigned zero free days. |
| Secondary infections | Identified based on positive blood, urine, and sputum cultures. Only infections occurring after administration of interleukin-6 Receptor Antagonists or steroids were considered. The definition of infection outcomes was following VIRUS Standard operating definitions |
| 28-day mortality | Defined as the time from hospital admission until death within 28 days, with censoring of alive patients at 28 days. |
| Acute respiratory distress syndrome (ARDS) | Classified according to the Berlin definition [1].  The PF ratio was calculated by dividing the arterial Po2 by the FiO2 at the same time. If the FiO2 was not available, the lowest FiO2 was used followed by the highest FiO2. |
| Critical COVID-19 | Defined by the WHO criteria for ARDS, sepsis, septic shock or other conditions that would normally require the provision of life-sustaining therapies, such as mechanical ventilation (invasive or non-invasive) or vasopressor therapy [2,3]. |
| Severe covid-19 | Defined by any of the following: oxygen saturation <90% on room air, respiratory rate >30 breaths per minute in adults, signs of severe respiratory distress (accessory muscle use, inability to complete full sentences) [3]. |
| ICU and hospital length of stay | the interval between the ICU or hospital admission date to the ICU or hospital  discharge date was calculated in days |

**References** :

(1) ARDS Definition Task Force, Ranieri VM, Rubenfeld GD, Thompson BT, Ferguson ND, Caldwell E, Fan E, Camporota L, Slutsky AS. Acute respiratory distress syndrome: the Berlin Definition. JAMA. 2012 Jun 20;307(23):2526-33. PMID: [22797452](https://www.ncbi.nlm.nih.gov/pubmed/22797452)

(2) Alhazzani W, Evans L, Alshamsi F, Møller MH, Ostermann M, Prescott HC, Arabi YM et al A. Surviving Sepsis Campaign Guidelines on the Management of Adults With Coronavirus Disease 2019 (COVID-19) in the ICU: First Update. Crit Care Med. 2021 Mar 1;49(3):e219-e234. doi: 10.1097/CCM.0000000000004899

(3) Rochwerg B, Agarwal A, Siemieniuk R A, Agoritsas T, Lamontagne F, Askie L et al. A living WHO guideline on drugs for covid-19 BMJ 2020; 370 :m3379 doi:10.1136/bmj.m3379

**Table S3: Distribution of baseline covariates across groups before adjustment**

|  | **Steroids** | **IL6-R antagonists** | **Both** | **P** |
| --- | --- | --- | --- | --- |
|  | **N=589** | **N=170** | **N=101** |  |
| **Sex** |  |  |  | 0.39 |
| Female | 205 (34.8) | 49 (28.8) | 34 (33.7) |  |
| Male | 384 (65.2) | 119 (70) | 70 (66.3) |  |
| **Age, mean (SD)** | 61.1 (14.3) | 57.4 (14.7) | 62.1 (13.2) | 0.01 |
| **Therapeutic anticoagulation** |  |  |  | 0.24 |
| No | 455 (77.2) | 136 (80) | 75 (74.3) |  |
| Yes | 134 (22.8) | 32 (20) | 29 (25.7) |  |
| **Ethnic group** |  |  |  | <0.001 |
| Hispanic | 112 (19) | 48 (28.2) | 22 (21.7) |  |
| Non-Hispanic | 263 (44.7) | 80 (47.1) | 55 (54.4) |  |
| **Weight (kg) at Hospital Admission, mean (SD)** | 90.1 (27.7) | 96.9 (28) | 95.3 (28.5) | 0.01 |
| **Cardiovascular disease** | 130 (22.1) | 16 (9.4) | 23 (22.7) | 0.001 |
| **Asthma/COPD** | 102 (17.3) | 19 (11.1) | 29 (28.7) | 0.002 |
| **Hydroxychloroquine** | 87 (14.8) | 87 (51.2) | 41 (40.6) | <0.001 |
| **Antiviral** | 474 (80.5) | 159 (93.5) | 86 (85.1) | <0.001 |
| **Azithromycin** | 204 (34.6) | 93 (54.7) | 50 (49.5) | <0.001 |
| **ARDS** |  |  |  | . |
| Mild (P:F 200-300) | 15 (2.7) | 4 (2.4) | 6 (5.9) |  |
| Moderate (P:F 100-199) | 65 (11.5) | 4 (2.4) | 8 (7.9) |  |
| Severe (P:F< 100) | 105 (18.6) | 12 (7.1) | 16 (15.8) |  |
| **Lowest FiO2, mean (SD)** | 0.57 (0.3) | 0.51 (0.3) | 0.55 (0.2) | 0.12 |

Data presented as n (%) unless otherwise specified

Abbreviation: ARDS: Acute respiratory distress syndrome ; COPD: Chronic obstructive pulmonary disease SD: standard deviation; P:F: ratio of arterial oxygen partial pressure (PaO2 in mmHg) to fractional inspired oxygen (FiO2)

**Table S4: Other Baseline characteristics of the total cohort**

|  | **Steroids**  **N=589** | **IL6-R antagonists**  **N=170** | **Both**  **N=101** | **P** | **N** |
| --- | --- | --- | --- | --- | --- |
| **Hospital Admission Source** |  |  |  | <0.001 | 858 |
| Home | 207 (35.3) | 42 (24.7) | 36 (35.6) |  |  |
| Hospital ED | 177 (30.2) | 92 (54.1) | 43 (42.6) |  |  |
| Nursing Home | 25 (4.26) | 2 (1.2) | 2 (1.9) |  |  |
| Other | 5 (0.85) | 1 (0.6) | 0 (0) |  |  |
| Outside ED | 30 (5.11) | 8 (4.7) | 3 (2.9) |  |  |
| Transfer from other Facility | 143 (24.4) | 25 (14.7) | 17 (16.8) |  |  |
| **Signs and symptoms** |  |  |  | 0.80 | 860 |
| Abdominal pain | 38 (6.5) | 5 (2.9) | 7 (6.9) | 0.20 | 860 |
| Ageusia (loss of taste sense) | 25 (4.24) | 5 (2.9) | 3 (2.9) | 0.82 | 860 |
| Loss of appetite | 51 (8.7) | 25 (14.7) | 13 (12.9) | 0.05 | 860 |
| Anosmia (loss of smell sense) | 19 (3.2) | 4 (2.4) | 6 (5.9) | 0.27 | 860 |
| Arthralgia | 11 (1.9) | 4 (2.4) | 6 (5.9) | 0.05 | 860 |
| Chest pain/tightness | 67 (11.4) | 16 (9.4) | 11 (10.9) | 0.77 | 860 |
| Chills/rigors | 84 (14.3) | 33 (19.4) | 15 (14.9) | 0.26 | 860 |
| Confusion/delirium | 49 (8.3) | 11 (6.5) | 7 (6.9) | 0.69 | 860 |
| Conjunctival congestion | 1 (0.17) | 1 (0.59) | 0 (0) | 0.53 | 860 |
| Dry cough | 283 (48) | 101 (59.4) | 58 (57.4) | 0.01 | 860 |
| Cough with sputum | 69 (11.7) | 25 (14.7) | 16 (15.8) | 0.37 | 860 |
| Diarrhea | 78 (13.2) | 44 (25.9) | 21 (20.8) | **<0.001** | 860 |
| Dizziness/lightheadedness | 31 (5.3) | 8 (4.7) | 5 (4.9) | 0.96 | 860 |
| Dyspnea/shortness of breath | 436 (74) | 133 (78.2) | 73 (72.3) | 0.45 | 860 |
| Fever | 370 (62.8) | 125 (73.5) | 72 (71.3) | **0.02** | 860 |
| Headache | 60 (10.2) | 23 (13.5) | 11 (10.9) | 0.47 | 860 |
| Hemoptysis | 7 (1.2) | 0 (0) | 0 (0) | 0.39 | 860 |
| Malaise | 91 (15.4) | 28 (16.5) | 23 (22.8) | 0.19 | 860 |
| Myalgia or fatigue | 439 (74.5) | 101 (59.4) | 62 (61.4) | **<0.001** | 860 |
| Nasal congestion/rhinorrhea | 25 (4.2) | 12 (7.06) | 10 (9.9) | **0.04** | 860 |
| Nausea and Vomiting | 81 (13.8) | 22 (12.9) | 15 (14.9) | 0.91 | 860 |
| Night sweat | 9 (1.5) | 1 (0.59) | 2 (1.9) | 0.60 | 860 |
| Seizure | 6 (1.02) | 1 (0.59) | 0 (0) | 0.85 | 860 |
| Sneezing | 1 (0.2) | 1 (0.59) | 0 (0) | 0.53 | 860 |
| Sore throat or throat pain | 33 (5.6) | 15 (8.8) | 11 (10.9) | 0.08 | 860 |
| Swollen neck/glands (lymphadenopathy) | 1 (0.2) | 0 (0) | 0 (0) | 1.00 | 860 |
| **Comorbidities** |  |  |  |  |  |
| Coronary heart diseases | 492 (83.5) | 161 (94.7) | 85 (84.2) | **0.001** | 860 |
| Hypertension | 251 (42.6) | 67 (39.4) | 45 (44.6) | 0.67 | 860 |
| Cardiac arrhythmias | 50 (8.5) | 5 (2.9) | 4 (3.9) | **0.02** | 860 |
| Congestive heart failure | 72 (12.2) | 10 (5.9) | 9 (8.9) | **0.05** | 860 |
| Valvular heart disease | 17 (2.9) | 1 (0.6) | 1 (0.9) | 0.18 | 860 |
| Chronic pulmonary disease not asthma | 59 (10) | 7 (4.1) | 16 (15.8) | **0.005** | 860 |
| Asthma physician diagnosed | 45 (7.6) | 12 (7.1) | 12 (11.9) | 0.31 | 860 |
| Pulmonary circulation disorder | 7 (1.2) | 1 (0.6) | 2 (1.9) | 0.55 | 860 |
| Chronic kidney disease | 58 (9.9) | 14 (8.24) | 12 (11.9) | 0.62 | 860 |
| Chronic dialysis | 12 (2.04) | 2 (1.18) | 3 (2.9) | 0.59 | 860 |
| Diabetes | 362 (61.5) | 111 (65.3) | 55 (54.5) | 0.21 | 860 |
| Hypothyroidism | 45 (7.6) | 6 (3.5) | 5 (4.9) | 0.13 | 860 |
| Liver disease | 20 (3.4) | 2 (1.2) | 2 (1.9) | 0.32 | 860 |
| Hepatitis B | 2 (0.34) | 1 (0.6) | 0 (0) | 0.68 | 860 |
| Hepatitis C | 11 (1.9) | 3 (1.8) | 2 (1.9) | 1.00 | 860 |
| peptic ulcer disease excluding bleeding | 6 (1.02) | 3 (1.8) | 2 (1.9) | 0.44 | 860 |
| solid tumor without metastasis | 26 (4.4) | 0 (0) | 8 (7.9) | **0.001** | 860 |
| hematologic malignancy | 7 (1.2) | 4 (2.4) | 1 (0.9) | 0.43 | 860 |
| metastatic cancer | 10 (1.7) | 0 (0) | 0 (0) | 0.12 | 860 |
| history of solid organ or bone marrow transplant | 1 (0.17) | 0 (0) | 2 (1.9) | 0.06 | 860 |
| HIV aids or other immunosuppression | 6 (1.02) | 4 (2.4) | 2 (1.9) | 0.24 | 860 |
| stroke or other neurological disorders | 47 (7.9) | 4 (2.4) | 5 (4.9) | **0.03** | 860 |
| Paralysis | 5 (0.9) | 1 (0.6) | 0 (0) | 1.00 | 860 |
| Rheumatoid arthritis collagen vascular disease | 9 (1.5) | 3 (1.8) | 1 (0.9) | 0.91 | 860 |
| Blood loss anemia | 0 (0) | 1 (0.6) | 0 (0) | 0.32 | 860 |
| Iron deficiency anemia | 13 (2.2) | 3 (1.8) | 2 (1.9) | 1.00 | 860 |
| Coagulopathy | 4 (0.68) | 3 (1.8) | 1 (0.9) | 0.32 | 860 |
| Malnutrition | 1 (0.17) | 0 (0) | 1 (0.9) | 0.26 | 860 |
| Obesity | 117 (19.9) | 60 (35.3) | 32 (31.7) | **<0.001** | 860 |
| Substance use disorder | 15 (2.6) | 2 (1.2) | 2 (1.9) | 0.63 | 860 |
| Depression | 37 (6.3) | 5 (2.9) | 10 (9.9) | 0.06 | 860 |
| Psychosis | 5 (0.9) | 1 (0.6) | 0 (0) | 1.00 | 860 |
| Dementia | 18 (3.1) | 3 (1.8) | 2 (1.9) | 0.76 | 860 |
| Obstructive sleep apnea | 33 (5.6) | 19 (11.2) | 11 (10.9) | **0.02** | 860 |
| Venous thromboembolism | 15 (2.6) | 4 (2.4) | 6 (5.9) | 0.19 | 860 |
| Dyslipidemia hyperlipidemia | 59 (10) | 6 (3.5) | 10 (9.9) | **0.03** | 860 |
| **Admission Diagnosis/Complications** |  |  |  |  |  |
| Acute Liver Injury | 22 (3.7) | 5 (2.9) | 6 (5.9) | 0.42 | 860 |
| Acute Myocardial Infarction | 9 (1.5) | 1 (0.6) | 0 (0) | 0.41 | 860 |
| Acute Renal Failure Requiring Hemofiltration | 23 (3.9) | 5 (2.9) | 8 (7.9) | 0.12 | 860 |
| Acute Renal Injury | 89 (15.1) | 31 (18.2) | 14 (13.9) | 0.54 | 860 |
| Bacteremia | 16 (2.7) | 4 (2.4) | 0 (0) | 0.29 | 860 |
| Bacterial Pneumonia | 59 (10) | 23 (13.5) | 8 (7.9) | 0.28 | 860 |
| Cardiac Arrest | 19 (3.2) | 1 (0.6) | 1 (0.9) | 0.09 | 860 |
| Cardiac Arrhythmia: Atrial Fibrillation | 10 (1.7) | 3 (1.8) | 4 (3.9) | 0.35 | 860 |
| Cardiac Arrhythmia: Heart Block | 2 (0.3) | 0 (0) | 1 (0.9) | 0.40 | 860 |
| Cardiac Arrhythmia: Ventricular Tachycardia | 8 (1.4) | 0 (0) | 2 (1.9) | 0.19 | 860 |
| Congestive Heart Failure / Cardiomyopathy | 13 (2.2) | 1 (0.6) | 3 (2.9) | 0.31 | 860 |
| Diabetic Ketoacidosis (DKA) | 2 (0.34) | 0 (0) | 0 (0) | 1.00 | 860 |
| Delirium / Encephalopathy | 48 (8.2) | 4 (2.4) | 3 (2.9) | **0.008** | 860 |
| Gastrointestinal Hemorrhage | 2 (0.3) | 1 (0.6) | 1 (0.9) | 0.38 | 860 |
| Hyperglycemia | 67 (11.4) | 22 (12.9) | 14 (13.9) | 0.71 | 860 |
| Hypoglycemia | 5 (0.9) | 0 (0) | 0 (0) | 0.63 | 860 |
| Meningitis/Encephalitis | 3 (0.5) | 0 (0) | 0 (0) | 1.00 | 860 |
| Myocarditis | 3 (0.5) | 0 (0) | 1 (0.9) | 0.53 | 860 |
| Pneumothorax | 3 (0.5) | 0 (0) | 2 (1.9) | 0.16 | 860 |
| Pleural Effusion | 6 (1.02) | 0 (0) | 1 (0.9) | 0.48 | 860 |
| Rhabdomyolysis / Myositis | 8 (1.4) | 0 (0) | 2 (1.9) | 0.19 | 860 |
| Seizure | 5 (0.9) | 0 (0) | 0 (0) | 0.63 | 860 |
| Shock | 72 (12.2) | 9 (5.3) | 9 (8.9) | **0.03** | 860 |
| Stroke | 8 (1.4) | 0 (0) | 0 (0) | 0.22 | 860 |
| **Pre-Hospital (Home) Medication** |  |  |  |  |  |
| Angiotensin-converting-enzyme Inhibitor | 97 (16.5) | 31 (18.2) | 20 (19.8) | 0.66 | 860 |
| Angiotensin receptor blocker | 68 (11.5) | 22 (12.9) | 13 (12.9) | 0.85 | 860 |
| Antiretroviral | 2 (0.3) | 2 (1.2) | 1 (0.9) | 0.23 | 860 |
| Antibiotics | 54 (9.2) | 11 (6.5) | 7 (6.9) | 0.46 | 860 |
| Anti-Diabetic | 44 (7.5) | 5 (2.9) | 13 (12.9) | **0.009** | 860 |
| Aspirin | 122 (20.7) | 31 (18.2) | 29 (28.7) | 0.11 | 860 |
| Chemotherapy currently or in the last 3 Months: | 12 (2.04) | 1 (0.6) | 2 (1.9) | 0.51 | 860 |
| Immunotherapy | 9 (1.5) | 0 (0) | 3 (2.9) | 0.11 | 860 |
| Inhaled corticosteroids | 54 (9.2) | 13 (7.7) | 13 (12.9) | 0.35 | 860 |
| NSAID/Ibuprofen | 49 (8.3) | 18 (10.6) | 10 (9.9) | 0.62 | 860 |
| Other anti-hypertensive agent (eg., beta blocker, calcium channel blocker, diuretic) | 211 (35.8) | 57 (33.5) | 37 (36.6) | 0.83 | 860 |
| Paracetamol/Acetaminophen | 81 (13.8) | 25 (14.7) | 22 (21.8) | 0.11 | 860 |
| Proton Pump Inhibitors | 76 (12.9) | 27 (15.9) | 20 (19.8) | 0.15 | 860 |
| Statins | 166 (28.2) | 58 (34.1) | 31 (30.7) | 0.32 | 860 |
| Anticoagulants | 46 (7.8) | 9 (5.3) | 10 (9.9) | 0.35 | 860 |
| Hydroxychloroquine (Plaquenil) | 5 (0.9) | 2 (1.2) | 1 (0.9) | 0.87 | 860 |
| **Social History** |  |  |  |  |  |
| Social history (current smoker) | 29 (4.9) | 7 (4.12) | 6 (5.94) | 0.80 | 860 |
| Social history (former smoker) | 119 (20.2) | 29 (17.1) | 25 (24.8) | 0.31 | 860 |
| Social history (vaping) | 0 (0) | 1 (0.59) | 0 (0.00) | 0.32 | 860 |
| Social history (alcohol use disorder) | 26 (4.4) | 6 (3.53) | 5 (4.95) | 0.81 | 860 |
| Social history (substance use disorder) | 14 (2.4) | 3 (1.76) | 1 (0.99) | 0.81 | 860 |
| Social history (unknown/not available) | 37 (6.3) | 5 (2.94) | 4 (3.96) | 0.19 | 860 |
| **Packs per day (PPD) patient smokes** |  |  |  | 0.09 | 49 |
| < ½ PPD (0.5 PPD) | 8 (21.1) | 0 (0) | 2 (33.3) |  |  |
| 1 PPD | 6 (15.8) | 0 (0) | 2 (33.3) |  |  |
| 1½ PPD (1.5 PPD) | 1 (2.6) | 0 (0) | 0 (0) |  |  |
| 2 PPD | 4 (10.5) | 1 (20) | 2 (33.3) |  |  |
| Unknown/Not Available | 19 (50) | 4 (80) | 0 (0) |  |  |
| Does/Did patient uses other tobacco products regularly? (Cigars) | 6 (1.02) | 0 (0) | 1 (0.9) | 0.48 | 860 |
| Does/Did patient uses other tobacco products regularly? (Pipes) | 1 (0.2) | 0 (0) | 1 (0.9) | 0.26 | 860 |
| Does/Did patient uses other tobacco products regularly? (Chewing tobacco) | 2 (0.3) | 0 (0) | 0 (0) | 1.00 | 860 |
| Does/Did patient uses other tobacco products regularly? (No other tobacco) | 9 (1.5) | 1 (0.6) | 3 (2.9) | 0.27 | 860 |
| Does/Did patient uses other tobacco products regularly? (Unknown/ Not available) | 19 (3.2) | 4 (2.4) | 2 (1.9) | 0.86 | 860 |
| **Type of Shock** |  |  |  | 0.49 | 80 |
| Cardiogenic shock | 5 (7.6) | 0 (0) | 0 (0) |  |  |
| Distributive shock | 59 (89.4) | 5 (83.3) | 8 (100) |  |  |
| Hypovolemic shock | 2 (3.03) | 1 (16.7) | 0 (0) |  |  |

Data presented as n (%) unless otherwise specified

Abbreviation: ARDS: Acute respiratory distress syndrome ; P:F: ratio of arterial oxygen partial pressure (PaO2 in mmHg) to fractional inspired oxygen (FiO2)

**Table S5: Ventilator and Radiology Characteristics of the included patients at days 0 and 1 of ICU stay**

|  | Day 0 | | | | Day 1 | | | |
| --- | --- | --- | --- | --- | --- | --- | --- | --- |
|  | Steroids | IL6-R antagonists | combination | P | Steroids | IL6-R antagonists | combination | P |
|  | *N=589* | *N=170* | *N=101* |  | *N=568* | *N=170* | *N=94* |  |
| **Performed diagnostics** |  |  |  |  |  |  |  |  |
| None | 31 (5.3) | 7 (4.1) | 7 (6.9) | 0.60 | 232 (40.8) | 49 (28.8) | 33 (35.1) | **0.02** |
| Chest X-ray | 467 (79.3) | 149 (87.6) | 89 (88.1) | **0.01** | 256 (45.1) | 108 (63.5) | 53 (56.4) | **<0.001** |
| CT Chest | 153 (26) | 25 (14.7) | 13 (12.9) | **<0.001** | 27 (4.75) | 6 (3.5) | 5 (5.3) | 0.73 |
| Lung US | 8 (1.4) | 0 (0) | 1 (0.9) | 0.31 | 2 (0.4) | 1 (0.6) | 0 (0) | 0.68 |
| Cardiac Echo | 26 (4.4) | 3 (1.8) | 2 (1.9) | 0.22 | 30 (5.3) | 4 (2.4) | 3 (3.2) | 0.27 |
| **infiltrates present in CXR** | 424 (91.4) | 136 (91.3) | 84 (94.4) | 0.62 | 244 (97.2) | 105 (97.2) | 53 (100) | 0.72 |
| **Interstitial pattern** | 108 (18.3) | 26 (15.3) | 24 (23.8) | 0.22 | 58 (10.2) | 18 (10.6) | 12 (12.8) | 0.76 |
| Opacities | 320 (54.3) | 103 (60.6) | 62 (61.4) | 0.20 | 194 (34.2) | 80 (47.1) | 37 (39.4) | 0.01 |
| Multifocal | 155 (26.3) | 50 (29.4) | 30 (29.7) | 0.62 | 79 (13.9) | 48 (28.2) | 23 (24.5) | **<0.001** |
| Bilateral | 336 (57) | 102 (60) | 63 (62.4) | 0.53 | 203 (35.7) | 90 (52.9) | 39 (41.5) | **<0.001** |
| Pleural effusion | 26 (4.4) | 6 (3.5) | 8 (7.9) | 0.22 | 15 (2.6) | 6 (3.5) | 4 (4.3) | 0.48 |
| **CT chest pattern**^¶^ |  |  |  |  |  |  |  |  |
| Ground Glass Opacity (GGO) | 104 (17.7) | 17 (10) | 13 (12.9) | 0.04 | 19 (3.4) | 6 (3.5) | 5 (5.3) | 0.57 |
| Crazy paving | 10 (1.7) | 1 (0.6) | 2 (1.9) | 0.57 | 2 (0.4) | 0 (0) | 1 (1.1) | 0.40 |
| Multifocal | 88 (14.9) | 14 (8.2) | 8 (7.9) | **0.02** | 11 (1.9) | 3 (1.8) | 2 (2.1) | 1 |
| Bilateral | 134 (22.8) | 18 (10.6) | 12 (11.9) | **<0.001** | 18 (3.2) | 6 (3.5) | 4 (4.3) | 0.76 |
| Lymphadenopathy (LAP) | 13 (2.2) | 1 (0.6) | 1 (0.9) | 0.40 | 3 (0.5) | 0 (0) | 0 (0) | 1 |
| Pleural effusion | 17 (2.9) | 2 (1.2) | 0 (0) | 0.12 | 7 (1.2) | 1 (0.6) | 0 (0) | 0.63 |
| Opacity | 49 (8.3) | 7 (4.1) | 5 (4.9) | 0.11 | 6 (1.1) | 0 (0) | 0 (0) | 0.43 |
| Consolidation | 27 (4.6) | 1 (0.6) | 0 (0) | 0.003 | 3 (0.5) | 1 (0.6) | 1 (1.1) | 0.78 |
| Pulmonary nodules | 2 (0.34) | 0 (0) | 0 (0) | 1.00 | 0 (0) | 0 (0) | 0 (0) | 1 |
| **Respiratory Rate- mean (SD)** | 21.0 (24.2) | 29.8 (61.5) | 17.9 (4.9) | 0.12 | 23.9 (36.2) | 34.2 (75.1) | 18.3 (5.6) | 0.07 |
| **Tidal volume (ml) mean (SD)** | 432 (121) | 439 (114) | 444 (97.8) | 0.83 | 432 (113) | 419 (108) | 442 (93.6) | 0.46 |
| **PEEP (cm H2O) mean (SD)** | 11.2 (3.6) | 12 (3.9) | 11.1 (4.3) | 0.36 | 11.8 (6.4) | 12.0 (3.8) | 11.5 (3.1) | 0.88 |
| **Ventilator mode** |  |  |  | **<0.001** |  |  |  | **<0.005** |
| Airway pressure release ventilation (APRV) | 6 (1.9) | 1 (2) | 6 (16.7) |  | 9 (2.5) | 1 (0.9) | 2 (3.7) |  |
| Pressure control (PC) | 49 (15.5) | 4 (8) | 8 (22.2) |  | 42 (11.5) | 5 (4.9) | 12 (22.2) |  |
| Pressure Support (PS) | 4 (1.3) | 1 (2) | 1 (2.8) |  | 4 (1.1) | 1 (0.9) | 1 (1.9) |  |
| Volume control (VC) | 206 (65) | 22 (44) | 16 (44.4) |  | 243 (66.8) | 63 (62.4) | 32 (59.3) |  |
| **Documented ventilator associated pneumonia (VAP)** | 15 (5.2) | 0 (0) | 4 (10.5) | **0.05** | 13 (3.9) | 3 (2.9) | 2 (3.7) | 1 |
| **Documented assessment of spontaneous breathing trial** |  |  |  | 0.32 |  |  |  | 0.46 |
| No | 145 (50.7) | 28 (54.9) | 18 (48.6) |  | 180 (54.9) | 55 (55) | 26 (48.1) |  |
| not indicated | 131 (45.8) | 22 (43.1) | 15 (40.5) |  | 118 (36) | 39 (39) | 26 (48.1) |  |
| Yes | 10 (3.5) | 1 (1.9) | 4 (10.8) |  | 30 (9.2) | 6 (6) | 2 (3.7) |  |

Data presented as n (%) unless otherwise specified

¶ percentage calculated based on available data for patients with computerized tomography (CT) chest scan

**Table S6: SOFA and APCHE-II scores at baseline**

|  | Steroids | IL-6 antagonists | Both | P | N |
| --- | --- | --- | --- | --- | --- |
|  | *N=446* | *N=105* | *N=73* |  |  |
| **SOFA Respiration:** |  |  |  | . | 622 |
| 0. >400 | 19 (4.3) | 10 (9.5) | 2 (2.8) |  |  |
| 1. < 400 (S:F 221-301), +/- Respiratory support | 25 (5.6) | 9 (8.6) | 6 (8.5) |  |  |
| 2. < 300 (S:F 142-220), +/- Respiratory support | 73 (16.4) | 19 (18.1) | 19 (26.8) |  |  |
| 3. < 200 (S:F 67-141) and Respiratory support | 165 (37) | 46 (43.8) | 26 (36.6) |  |  |
| 4. < 100 (S:F <67) and Respiratory support | 164 (36.8) | 21 (20) | 18 (25.4) |  |  |
| **SOFA coagulation (platelet):** |  |  |  | 0.41 | 582 |
| 0. >150 | 338 (83.5) | 86 (82.7) | 60 (82.2) |  |  |
| 1. < 150 | 47 (11.6) | 11 (10.6) | 13 (17.8) |  |  |
| 2. < 100 | 14 (3.5) | 6 (5.8) | 0 (0) |  |  |
| 3. < 50 | 5 (1.2) | 1 (0.9) | 0 (0) |  |  |
| 4. < 20 | 1 (0.3) | 0 (0) | 0 (0) |  |  |
| **SOFA cardiovascular (vasopressors):** |  |  |  | . | 583 |
| 0. No Hypotension | 233 (57.5) | 60 (57.1) | 40 (54.8) |  |  |
| 1. MAP < 70 mm Hg | 25 (6.2) | 21 (20) | 11 (15.1) |  |  |
| 2. Dopamine ≤ 5 or Dobutamine (any dose) | 1 (0.3) | 0 (0) | 1 (1.4) |  |  |
| 3. Dopamine > 5 or Epi/Norepi ≤0.1 | 70 (17.3) | 11 (10.5) | 11 (15.1) |  |  |
| 4. Dopamine > 15 or Epi/Norepi > 0.1 | 76 (18.8) | 13 (12.4) | 10 (13.7) |  |  |
| **SOFA GCS:** |  |  |  | 0.01 | 582 |
| 0. 15 | 176 (43.5) | 62 (59) | 38 (52.8) |  |  |
| 1. 13-14 | 37 (9.1) | 9 (8.6) | 4 (5.6) |  |  |
| 2. 10-12 | 30 (7.4) | 9 (8.6) | 6 (8.3) |  |  |
| 3. 6-9 | 73 (18) | 18 (17.1) | 16 (22.2) |  |  |
| 4. <6 | 89 (22) | 7 (6.7) | 8 (11.1) |  |  |
| **SOFA Liver (bilirubin):** |  |  |  | 0.16 | 569 |
| 0. < 1.2 | 335 (84.8) | 92 (89.3) | 64 (90.1) |  |  |
| 1. 1.2 -1.9 mg/dl .......... 20-32 micromole/L | 41 (10.4) | 7 (6.8) | 2 (2.8) |  |  |
| 2. 2.0 - 5.9 mg/dl ......... 33-101 micromole /L | 18 (4.6) | 3 (2.9) | 4 (5.6) |  |  |
| 3. 6.0-11.9 mg/dl ......... 102-204 micromole /L | 1 (0.3) | 1 (0.9) | 1 (1.4) |  |  |
| **SOFA Renal (creatinine or urine output):** |  |  |  | . | 583 |
| 0. < 1.2 mg/dl (110μmol/L) | 254 (62.7) | 73 (69.5) | 46 (63) |  |  |
| 1. 1.2 -1.9 mg/dl (110-170μmol/L) | 77 (19) | 21 (20) | 15 (20.5) |  |  |
| 2. 2.0 - 2.4 mg/dl (171-299μmol/L) | 28 (6.9) | 5 (4.8) | 6 (8.2) |  |  |
| 3. 2.5-4.9 mg/dl (300-440μmol/L) or urine output < 500 mL/day | 30 (7.4) | 5 (4.8) | 4 (5.5) |  |  |
| 4. > 5.0 mg/dl (>440μmol/L) or urine output < 200 mL/day | 16 (3.9) | 1 (0.9) | 2 (2.7) |  |  |
| **Calculated APACHE II (First 24 hours in ICU that is Day 1 ICU)** |  |  |  | <0.001 | 117 |
| No | 23 (46.9) | 4 (9.1) | 6 (25) |  |  |
| Yes | 26 (53.1) | 40 (90.9) | 18 (75) |  |  |
| APACHE II (First 24 hours) score, mean (SD) | 18.1 (7.1) | 18.2 (6.9) | 18.8 (7.9) | 0.94 | 83 |

Data presented as n (%) unless otherwise specified

**Table S7: ICU support and therapeutic interventions at days 0 of ICU admission after IPTW and matching**

|  | **Steroids** | **IL6-R antagonists** | **Both** | **P** | **SMD** |
| --- | --- | --- | --- | --- | --- |
| n¶ | 804.1 | 582.4 | 689.6 |  |  |
| **Code status change** |  |  |  | 0.62 | 0.137 |
| N/A | 17 ( 2.4) | 23 (4.0) | 11 (1.7) |  |  |
| No | 690 ( 94.9) | 531 (94.7) | 624 (94.8) |  |  |
| Yes | 20 ( 2.8) | 7 ( 1.3) | 23 ( 3.5) |  |  |
| **Code change** |  |  |  | 0.14 | 1.284 |
| Changed to 'no chest compression' (no CPR) -DNR | 12 ( 58.6) | 3 ( 40.1) | 0 ( 0) |  |  |
| Changed to both- DNI-DNR | 3 ( 12.8) | 0 ( 0) | 4 ( 18.0) |  |  |
| Changed to full support (full code) | 6 ( 28.6) | 4 ( 59.9) | 19 ( 82.0) |  |  |
| **Neuromuscular blocker** |  |  |  | 0.08 | 0.423 |
| Atracurium | 10 (1.6) | 6 (1.6) | 30 (6.4) |  |  |
| Cisatracurium | 75 (12.4) | 24 (6.2) | 26 (5.4) |  |  |
| Pancuronium | 4 ( 0.6) | 0 (0) | 0 (0) |  |  |
| Rocuronium | 117 ( 19.3) | 125 (32.6) | 125 (26.3) |  |  |
| Succinylcholine | 4 ( 0.6) | 5 ( 1.2) | 12 (2.5) |  |  |
| Vecuronium | 16 ( 2.7) | 12 ( 3.1) | 0 (0) |  |  |
| None | 370 ( 60.8) | 207 (54.0) | 277 (58.5) |  |  |
| **Stress ulcer prophylaxis** | 609 ( 77.6) | 367 (66.9) | 462 (70.2) | 0.14 | 0.159 |
| Esomeprazole | 22 ( 3.0) | 0 (0) | 0 (0) |  |  |
| Famotidine | 141 (19.4) | 110 (19.6) | 122 (18.7) |  |  |
| Lansoprazole | 20 ( 2.7) | 25 ( 4.5) | 39 (5.9) |  |  |
| Omeprazole | 92 (12.6) | 63 ( 11.2) | 98 (14.9) |  |  |
| Pantoprazole | 163 (22.4) | 58 (10.3) | 175 (26.7) |  |  |
| Ranitidine | 46 ( 6.3) | 0 ( 0.0) | 4 (0.6) |  |  |
| None | 210 (28.9) | 223 ( 39.8) | 176 (26.9) |  |  |
| Not Indicated | 30 ( 4.1) | 78 ( 14.0) | 41 (6.2) |  |  |
| Stress ulcer prophylaxis route (IV) | 325 (66.7) | 162 ( 62.4) | 287 (65.7) | 0.86 | 0.06 |
| Anticoagulant | 687 ( 85.4) | 494 ( 84.7) | 549 (79.6) | 0.36 | 0.102 |

Data presented as n (%) unless otherwise specified. N (%) may differ due to approximation

¶ Each individual gets its own weight that is used for further analysis. Multiplication by these weights can usually results in decimals

**Abbreviation:** CPR: Cardiopulmonary resuscitation; DNR: Do-not-resuscitate order; DNI: Do Not Intubate; IV: intravenous

**Table S8: Distribution of Anakinra and Remdesivir doses**

| Anakinra doses | N (%) |
| --- | --- |
| 0 | 851 (99) |
| 1 | 1 (0.1) |
| 2 | 3 (0.4) |
| 3 | 2 (0.2) |
| 4 | 2 (0.2) |
| 5 | 1 (0.1) |
| Remdesivir doses |  |
| 0 | 737 (85.7) |
| 1 | 24 (2.8) |
| 2 | 22 (2.6) |
| 3 | 21 (2.4) |
| 4 | 27 (3.1) |
| 5 | 14 (1.6) |
| 6 | 2 (0.2) |
| 7 | 2 (0.2) |
| 8 | 3 (0.4) |
| 9 | 1 (0.1) |
| 10 | 6 (0.7) |
| 11 | 1 (0.1) |

Data presented as n (%) unless otherwise specified

**Table S9: When was the patient admitted to ICU with respect to hospital admission day.**

|  | N (% of patients admitted to ICU) | | |
| --- | --- | --- | --- |
| Hospital day | **Steroid (n=589)** | **IL6-R antagonists (n= 170)** | **Both (n=101)** |
| Day 0 | 437 (74.2)^a^ | 96 (56.5) ^a^ | 59 (58.4) ^a^ |
| Day 1 | 71 (12.1) | 49 (28.8) | 31 (30.7) |
| Day 2 | 21 (3.57) | 9 (5.29) | 2 (1.98) |
| Day 3 | 27 (4.58) | 7 (4.12) | 5 (4.95) |
| Day 4 | 12 (2.04) | 1 (0.60) | 3 (2.97) |
| Day 5 | 2 (0.34) | 4 (2.35) | 0 (0.00) |
| Day 6 | 1 (0.17) | 1 (0.60) | 0 (0.00) |
| Day 7 | 13 (2.21) | 1 (0.60) | 2 (1.98) |
| Day 8 | 3 (0.51) | 0 (0.00) | 1 (0.99) |

^a^ For many patients, the progression to critical illness occurs in a short period of time and this percentage also included patients who transferred from other hospitals

**Table S10:**  **Clinical outcomes before matching**

|  | **Steroids** | **IL6-R antagonists** | **Both** | **P overall** |
| --- | --- | --- | --- | --- |
|  | **N=589** | **N=170** | **N=101** |  |
| **ICU discharge status** |  |  |  | <0.001 |
| Alive | 296 (50.3) | 118 (69.4) | 51 (50.4) |  |
| Deceased | 287 (48.7) | 50 (30.6) | 53 (49.5) |  |
| **Hospital discharge status** |  |  |  | <0.001 |
| Alive | 294 (49.9) | 119 (70) | 47 (46.5) |  |
| Deceased | 295 (50.1) | 51 (30) | 54 (53.5) |  |
| **Ventilation free days, median IQR** | 18 [10;22] | 16.2 [7.50;21.4] | 14 [6.4;21] | 0.03 |
| **Duration of mechanical ventilation**  **(Survivors only)** | 10.42 [6; 20.5] | 13.75 [7.8; 22.1] | 14.2 [9.6; 20.8] | 0.16 |
| **Total ICU length of Stay, median IQR** | 13.8 [8.00;22.0] | 13.4 [8.14;23.8] | 17.0 [9.38;24.3] | 0.08 |
| **Total ICU length of Stay in survivor, median IQR**  (Survivors only) | 15.2 [9.00;25.0] | 14.0 [8.89;24.0] | 18.0 [13.1;25.6] | 0.22 |
| **Total hospital length of Stay, median IQR** | 18 [10.6;28.4] | 19 [11.0;31] | 23.9 [13.1;33] | 0.05 |
| Statistical analysis was performed using Chi-square test of independent for ICU discharge and Kruskal-Wallis test for continuous variables  Data presented as n (%) unless otherwise specified | | | | |

**Table S11: Linear regression analysis for ventilation-free days after adjusting for covariates stratified by steroid dose**

| *Predictors* | *ß* | *95% CI* | *P* |
| --- | --- | --- | --- |
| **Group** |  |  |  |
| **IL-6 antagonist** | *Reference* |  |  |
| **Steroid Low-dose** | 0.62 | -1.54 – 2.78 | 0.57 |
| **Steroid High-dose** | -1.19 | -3.85 – 1.47 | 0.38 |
| Age | -0.01 | -0.07 – 0.04 | 0.59 |
| Weight | -0.02 | -0.05 – 0.00 | 0.07 |
| Lowest FiO2 | 3.20 | 1.26 – 8.27 | **0.02** |
| **Gender** |  |  |  |
| Female | *Reference* |  |  |
| Male | -1.09 | -2.58 – 0.40 | 0.15 |
| **Neuromuscular blocker** |  |  |  |
| Yes | *Reference* |  |  |
| No | 2.87 | 0.18 – 5.55 | **0.04** |
| **Vasopressors** |  |  |  |
| Yes | *Reference* |  |  |
| No | -1.69 | -4.17 – 0.79 | 0.18 |
| **Therapeutic anticoagulation** |  |  |  |
| No | *Reference* |  |  |
| Yes | 2.02 | 0.26 – 3.78 | **0.03** |
| **Any anticoagulation** |  |  |  |
| Yes | *Reference* |  |  |
| No | -1.36 | -3.45 – 0.73 | 0.20 |
| **Ethnicity** |  |  |  |
| Hispanic | *Reference* |  |  |
| Non-Hispanic | 0.67 | -1.20 – 2.54 | 0.48 |
| **Hydroxychloroquine** |  |  |  |
| Yes | *Reference* |  |  |
| No | 1.36 | -0.57 – 3.30 | 0.17 |
| **Antivirals** |  |  |  |
| Yes | *Reference* |  |  |
| No | 0.04 | -1.87 – 1.95 | 0.97 |
| **Azithromycin** |  |  |  |
| Yes | *Reference* |  |  |
| No | -0.28 | -1.85 – 1.30 | 0.73 |
| **ARDS grade** |  |  |  |
| No | *Reference* |  |  |
| Mild (P:F 200-300) | -3.47 | -7.78 – 0.84 | 0.11 |
| Moderate (P:F 100-199) | -1.27 | -3.96 – 1.41 | 0.35 |
| Severe (P:F< 100) | -1.63 | -3.88 – 0.62 | 0.16 |
| **CVD** |  |  |  |
| No | *Reference* |  |  |
| Yes | -0.97 | -2.79 – 0.85 | 0.29 |
| **Asthma/COPD** |  |  |  |
| No | *Reference* |  |  |
| Yes | 0.40 | -1.45 – 2.25 | 0.67 |

The normality of residuals was inspected to ensure good model fit for the linear regression model.

ARDS = Acute respiratory distress syndrome; CI= confidence interval; CVD = cardiovascular diseases; COPD = Chronic obstructive pulmonary disease; P:F = ratio of arterial oxygen partial pressure (PaO2 in mmHg) to fractional inspired oxygen (FiO2);  *ß=* the log of incident rate ratio. If 0, then it is not significant

**Table S12:**  **Association between medication use and mortality after adjustment stratified by steroid dose**

The model was adjusted for the same variables used for IPTW.

Abbreviation: OR: odds ratio; CI: confidence interval; IPTW: Inverse probability weighting

| *Predictors* | *Adj Odds Ratio* | *95% CI* | *p* |
| --- | --- | --- | --- |
| **IL-6R antagonist** | *Reference* |  |  |
| **Steroid Low-dose** | 1.28 | 0.66 – 2.51 | 0.46 |
| **Steroid High-dose** | 1.73 | 0.75 – 4.04 | 0.20 |
| Age (1 year increase) | 1.05 | 1.03 – 1.07 | **<0.001** |
| Weight (1 Kg increase) | 1.00 | 0.99 – 1.01 | 0.90 |
| Lowest FiO2 | 3.20 | 1.26 – 8.27 | **0.02** |
| **Gender** |  |  |  |
| Female | *Reference* |  |  |
| Male | 1.32 | 0.82 – 2.15 | 0.26 |
| **Neuromuscular blocker** |  |  |  |
| Yes | *Reference* |  |  |
| No | 0.87 | 0.40 – 1.92 | 0.73 |
| **Vasopressors** |  |  |  |
| Yes | *Reference* |  |  |
| No | 0.88 | 0.42 – 1.82 | 0.72 |
| **Therapeutic anticoagulation** |  |  |  |
| No | *Reference* |  |  |
| Yes | 1.41 | 0.80 – 2.46 | 0.23 |
| **Any anticoagulation** |  |  |  |
| Yes | *Reference* |  |  |
| No | 0.60 | 0.31 – 1.16 | 0.13 |
| **Ethnicity** |  |  |  |
| Hispanic | *Reference* |  |  |
| Non-Hispanic | 1.41 | 0.79 – 2.55 | 0.25 |
| **Hydroxychloroquine** |  |  |  |
| Yes | *Reference* |  |  |
| No | 1.16 | 0.62 – 2.16 | 0.64 |
| **Antivirals** |  |  |  |
| Yes | *Reference* |  |  |
| No | 0.82 | 0.44 – 1.54 | 0.53 |
| **Azithromycin** |  |  |  |
| Yes | *Reference* |  |  |
| No | 1.32 | 0.78 – 2.22 | 0.30 |
| **ARDS grade** |  |  |  |
| No | *Reference* |  |  |
| Mild (P:F 200-300) | 0.42 | 0.07 – 2.20 | 0.32 |
| Moderate (P:F 100-199) | 0.61 | 0.25 – 1.46 | 0.27 |
| Severe (P:F< 100) | 0.98 | 0.48 – 1.97 | 0.95 |
| **Cardiovascular diseases** |  |  |  |
| No | *Reference* |  |  |
| Yes | 2.27 | 1.29 – 4.08 | **0.01** |
| **Asthma/COPD** |  |  |  |
| No | *Reference* |  |  |
| Yes | 0.94 | 0.51 – 1.72 | 0.84 |

**Table S13: Other documented complication during hospitalization after adjusting for baseline covariates**

|  | Steroids | IL6-R antagonists | Both | p |
| --- | --- | --- | --- | --- |
| N¶ | 804.1 | 582.4 | 689.6 |  |
| Anemia | 155 (19.2) | 145 (24.9) | 154 (22.3) | 0.50 |
| Bed ulcers | 40 ( 4.9) | 23 ( 3.9) | 28 ( 4.1) | 0.88 |
| Cardiomyopathy | 27 ( 3.4) | 13 ( 2.3) | 27 ( 3.9) | 0.73 |
| Secondary infection | 125 (15.5) | 82 (14.1) | 93 (13.5) | 0.88 |
| Congestive heart failure | 34 ( 4.3) | 15 ( 2.6) | 12 ( 1.8) | 0.40 |
| Endocarditis | 0 ( 0) | 6 ( 1.1) | 0 (0) | 0.24 |
| Gastrointestinal hemorrhage | 29 ( 3.6) | 17 ( 2.9) | 24 (3.5) | 0.92 |
| Hepatomegaly | 4 ( 0.5) | 0 ( 0) | 0 (0) | 0.41 |
| High bnp nt pro bnp | 34 ( 4.2) | 29 ( 4.9) | 34 (4.9) | 0.91 |
| Hyperglycemia | 200 (24.9) | 161 (27.7) | 206 (29.8) | 0.62 |
| Hypoglycemia | 31 ( 3.9) | 6 ( 1.0) | 27 ( 3.9) | 0.25 |
| Meningitis encephalitis | 15 ( 1.8) | 3 ( 0.5) | 0 (0) | 0.07 |
| Pancreatitis | 2 ( 0.2) | 0 ( 0) | 0 (0) | 0.67 |
| Pleural effusions | 54 ( 6.7) | 63 (10.7) | 24 ( 3.5) | 0.07 |
| Pleurisy | 2 ( 0.2) | 3 ( 0.4) | 0 (0) | 0.47 |
| Respiratory failure (ARDS) | 520 (64.7) | 315 (54.1) | 460 (66.7) | 0.11 |
| Rhabdomyolysis myositis | 20 ( 2.5) | 20 ( 3.3) | 11 ( 1.6) | 0.57 |
| Seizure | 17 ( 2.2) | 11 ( 1.9) | 7 ( 1.0) | 0.69 |
| Septic shock | 300 (37.3) | 279 (48.0) | 307 (44.4) | 0.19 |
| Splenomegaly | 3 ( 0.4) | 4 ( 0.7) | 0 (0) | 0.30 |
| St elevations in ECG | 8 ( 1.0) | 9 ( 1.6) | 0 (0) | 0.09 |
| Stroke or cerebrovascular accident | 25 ( 3.1) | 16 ( 2.8) | 10 ( 1.4) | 0.71 |

Data presented as n (%) unless otherwise specified. N (%) may differ due to approximation

¶ Each individual gets its own weight that is used for further analysis. Multiplication by these weights can usually results in decimals

**Table S14::** Post-hoc analysis for changes in selected biomarkers values using linear mixed modelling up to 28 days

Linear mixed modelling was used to assess whether the change in lab values across time was significantly different between groups. The interaction between time and medication was included in the model. The same variables used for IPTW were used for the analysis. Only results for the interaction are shown.

These results represent the change in CRP units based on the change of covariates. In CRP for example, the coefficients for IL-6 antagonist represent the expected change in CRP units with each day in the IL-6 group relative to steroid group. Thus, the -0.98 means that the reduction in CRP per day is higher by 0.98 units in the IL-6 antagonist compared to the steroid group.

|  | ß | SE | t | p |
| --- | --- | --- | --- | --- |
| **CRP (n = 247)** |  |  |  |  |
| IL-6R antagonist | -0.98 | 1.8 | -0.55 | 0.58 |
| Both | -3.39 | 1.64 | -2.07 | **0.04** |
| **LDH (n = 205)** |  |  |  |  |
| IL-6R antagonist | 0.01 | 0.12 | 0.12 | 0.91 |
| Both | 0.02 | 0.12 | 0.19 | 0.85 |
| **Ferritin (n = 220)** |  |  |  |  |
| IL-6R antagonist | -51.65 | 24.67 | -2.09 | **0.04** |
| Both | -32.19 | 19.77 | -1.63 | 0.10 |
| N represents the number of available patients for the analysis  Steroids alone group was a reference group | | | | |

**Abbreviation**: CRP= C-reactive protein; IL6-R antagonists= Interleukin-6 receptor antagonists; IPTW= Inverse Probability Treatment Weighting, LDH= Lactate Dehydrogenase

**Table S15: Linear mixed modelling of PF ratio**

Linear mixed model and linear marginal trends were used to assess changes in the PF ratio after adjusting for the same covariates and showed that the use of IL-6RA or combination therapy was not associated with significantly different changes in the PF ratio compared with the use of steroids alone.

| *Predictors* | ß | *95 %CI* | *P* |
| --- | --- | --- | --- |
| Steroids | *Reference* |  |  |
| IL-6R antagonists | 8.68 | -6.67 – 24.02 | 0.27 |
| Both | -1.51 | -18.30 – 15.28 | 0.86 |
| ICU day | 1.30 | 0.42 – 2.18 | **0.004** |
| sex: Male | -7.64 | -17.67 – 2.38 | 0.14 |
| Age (1 year increase) | -0.30 | -0.67 – 0.07 | 0.11 |
| Weight(kg)at Hospital Admission (1 Kg increase) | -0.31 | -0.48 – -0.13 | **0.001** |
| therapeutic anticoagulation: Yes | -6.12 | -15.92 – 3.67 | 0.22 |
| Any anticoagulation: Yes vs. No | -0.56 | -14.92 – 13.80 | 0.94 |
| ethnic group: Non-Hispanic | -2.46 | -15.04 – 10.12 | 0.70 |
| med hydroxychloroquine: No | -6.73 | -17.76 – 4.29 | 0.23 |
| med antiviral: No | 5.57 | -5.01 – 16.15 | 0.30 |
| med azithromycin: No | 10.74 | 1.44 – 20.04 | **0.02** |
| ARDS: Mild(P:F 200-300) | 15.11 | -14.74 – 44.97 | 0.32 |
| ARDS: Moderate(P:F 100-199) | 3.82 | -14.70 – 22.34 | 0.69 |
| ARDS: Severe(P:F<100)¶ | -33.64 | -47.96 – -19.32 | **<0.001** |
| CVD: Yes | 6.32 | -6.09 – 18.72 | 0.32 |
| Asthma/COPD: Yes | -5.93 | -19.29 – 7.43 | 0.38 |
| Respiratory failure: No | 0.99 | -11.34 – 13.31 | 0.88 |
| IL-6 antagonists x ICU day | 1.12 | -1.30 – 3.55 | 0.36 |
| Both meds x ICU day * | 0.96 | -1.06 – 2.97 | 0.35 |

**ß:** estimate represents average change in PFO2 for each 1 unit change in the predictor i.e. the increase in the PF ratio for each 1 unit increase in the predictor for IL-6R antagonists, steroids, and combination group.

Linear mixed modelling is similar to linear regression. The coefficients represent the change in the outcome for each 1 unit change if the predictor is continuous. If the predictor is categorical, it represents the average change between the specified level and the reference category

¶The -33.64 means that the average PF ratio is lower by 33.64 in patients with severe ARDS compared to patients with no ARDS

* This represents the average change in PF ratio per day in each of these groups for each 1 day increase in the ICU stay. for example, the 1.12 means that the average increase in the PF ration (per day) in patients who received both meds is higher by 1.12 units compared to patients who received steroids

Linear mixed modeling considers clustering within patients. Time was included as the main effect of the model. The same covariates used for MSM were used in the mixed model to ensure consistency. PF ratio data were available for 482 patients. Estimated marginal trends were used to visualize the change in the ratio after adjusting for baseline factors (e.g., age and sex) and time-varying covariates, such as the use of anticoagulation. Missing values were not imputed. The interaction between time and medication use was also included in the model to assess whether the change in the PF ratio was significantly different between the groups.

**Table S16:** Post-hoc analysis for the ventilator-free days outcome stratified by time of administration

| **Steroids** | | | |
| --- | --- | --- | --- |
| Outcome | Early | Late | P |
| VFD | Ref | 0.92 (0.74 – 1.13) | 0.418 |
| **IL-6 antagonists** | | | |
| VFD | Ref | 0.73 (0.53 – 1.01) | 0.06 |
| Results for VFD represent aIRR < 1 is favorable for control/reference group (early)  **Abbreviation**: aIRR= adjusted incident rate ratio, aOR= adjusted odds ratio, IL6-R antagonists= Interleukin-6 receptor antagonists; VFDs = ventilation free days | | | |

This analysis compared early administration (which was defined as receiving IL-6RA or steroids at days 0 or 1) versus late otherwise. The results were more favorable with early administration of steroids and IL-6RA as compared with late administration. VFD (<1 is favorable for reference group which is early group).

**Figure S1:.** Adjusted survival curves for competing risk models for (A) mortality (B) discharge

Risk regression models for survival endpoints in the presence of competing risks were fitted using binomial regression based on a time sequence of binary event status variables. The same predictors used for marginal structured model were used in the cause-specific Cox regression analysis.

| **Figure S2:** Unweighted and weighted Kaplan–Maier estimates for association between treatment ICU LOS   |
| --- |
| **Figure S3:** Unweighted and weighted Kaplan–Maier estimates for association between treatment and hospital LOS  |

**Figure S4: Estimated marginal trend for the change in PF ratio across groups.**


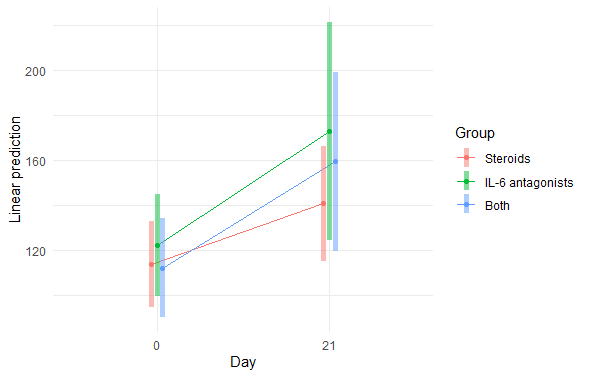


**Figure S5: Post-hoc analysis for the clinical outcomes ( ventilator-free days and mortality) stratified by age (> 60 vs. ≤ 60)**


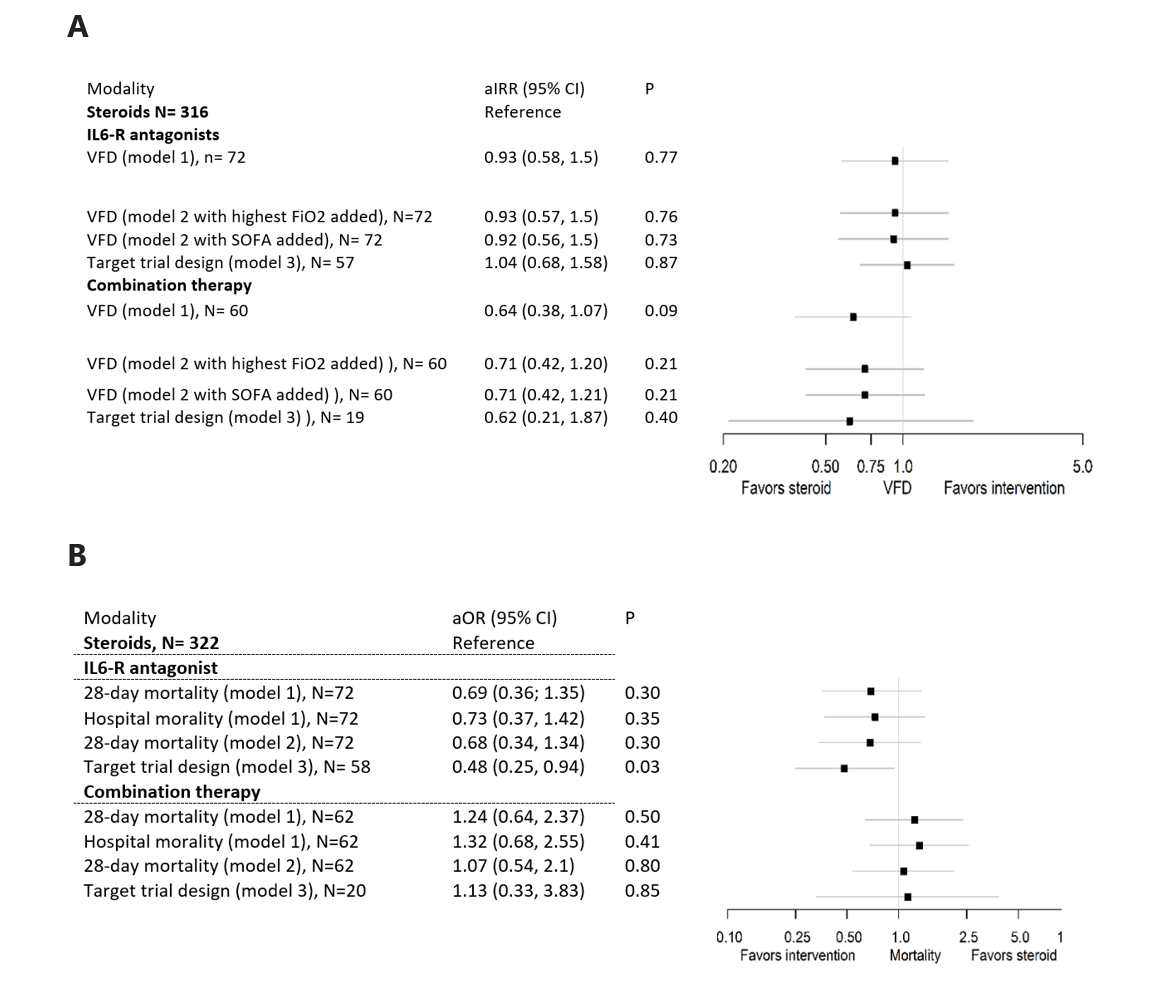


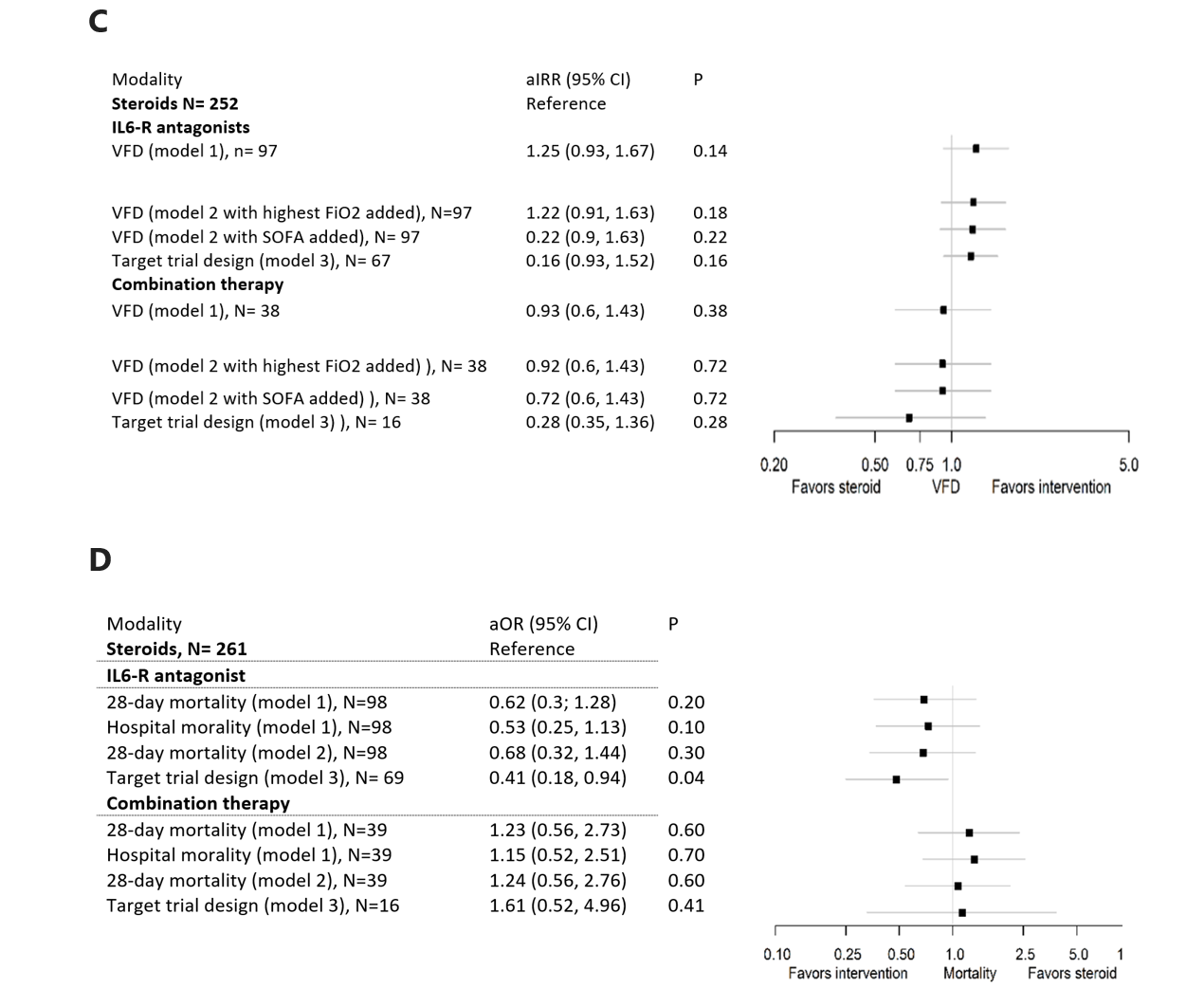


**Figure legend.** Association between treatment modality, ventilator-free days, and 28-day mortality stratified by age . (A-B) ventilator-free days and 28-day mortality for patients > 60 years. (C-D) ventilator-free days and 28-day mortality for patients ≤ 60 years. Adjusted incident rate ratio (aIRR) was used for VFD (>1 is favorable for intervention), Adjusted odds ratio (aOR) was used for 28-day mortality (< 1 is favorable for intervention)

**Abbreviation**: aIRR= adjusted incident rate ratio, aOR= adjusted odds ratio, FiO2 = fractional inspired oxygen concentration, IL6-R antagonists= Interleukin-6 receptor antagonists, SOFA= Sequential Organ Failure Assessment; VFDs = ventilation free days.

As shown by the point estimates, younger patients perhaps had more favorable outcomes with IL-6RA in VFDs and mortality compared with older population.
