# Supplementary material for: Clinical characteristics and outcomes of critically ill mechanically ventilated COVID-19 patients receiving interleukin-6 receptor antagonists and corticosteroid therapy: a preliminary report from a multinational registry
Source: Eur J Med Res. 2021 Oct 2;26:117. doi: 10.1186/s40001-021-00591-x (PMC8487342; doi:10.1186/s40001-021-00591-x)
Supplement: Supplementary file 2 — Additional file 2. Details for marginal structural model (MSM). [file 40001_2021_591_MOESM2_ESM.docx]

International System of Units (SI) were used to represent laboratory data unless otherwise specified. The data were checked for integrity and outliers in the laboratory data (numbers > the 99th percentile) were removed based on clinical judgment and whether the extreme values were clinically plausible.

**Marginal structural modeling (MSM):** allows for proper adjustment of time-dependent confounding variables, immortal time bias, and indication bias. Immortal time bias refers to the requirement that patients survive long enough to receive the intervention of interest, leading to a potential incorrect overestimation of a positive treatment effect. Indication bias from time-varying confounding variables refers to having an association related to the indication of the intervention that evolves throughout the course of an illness.

MSM using propensity scores were used to compare the primary and secondary outcomes between the three regimens after adjusting for non-time and time-varying covariates [1]. Because corticosteroid and Interleukin-6 receptor antagonists (IL-6RA) therapy often was not started at the time of ICU admission, but rather during the course of the disease on the basis of a change in the patient condition, we performed marginal structural model analysis with inverse probability of treatment weighting to account for time-varying confounders that are likely to influence the corticosteroid and IL-6RA therapy initiation and at the same time are likely to be correlated with the clinical outcome.

For dichotomous outcomes, the corresponding mixed effects model, namely a mixed effects logistic regression gives fixed effects coefficients that have an interpretation conditional on the random effects. Most often, this is not the interpretation we want. The Generalized Estimating Equations (GEE) approach does give coefficients with a marginal / population-averaged interpretation.

However, an additional practical point that also need to consider is missing data and almost always we have to deal with incomplete data. With regard to this point, mixed models give valid results under the less stringent missing at random assumption compared to the (standard not weighted) GEE that give valid results under the less realistic missing completely at random assumption.

Taking both points (i.e., interpretation and missing data) into account, would most often like to fit a mixed model to be more protected for the missing data but want to obtain parameters that have a population averaged interpretation. An early solution towards this direction was the marginalized mixed models proposed by Heagerty (2), but, in general, these are computationally intensive to fit. A more recent approach that seems to solve the problem has been proposed by Hedeker et al (3). This is implemented in the function marginal_coefs() of the R package GLMMadaptive.

The fixed effects estimates in mixed models with nonlinear link functions have an interpretation conditional on the random effects. However, often we wish to obtain parameters with a marginal / population averaged interpretation, which leads many researchers to use generalized estimating equations, and dealing with potential issues with missing data. Nonetheless, recently Hedeker et al. have proposed a nice solution to this problem. Their approach is implemented in function marginal_coefs(). For example, for model fm we obtain the marginalized coefficients.

The function calculates the marginal log odds ratios in our case (because we have a binary outcome) using a Monte Carlo procedure with number of samples determined by the M argument. Standard errors for the marginalized coefficients are obtained by setting std_errors = TRUE in the call to marginal_coefs(), and require a double Monte Carlo procedure for which argument K comes also into play.

Generalized estimating equations (GEE) were constructed to evaluate the impact of the treatment (t) on the outcome (d) and to handle the time-varying covariate (v) in the panel dataset. Since the outcome was a continuous variable, a generalized linear Gaussian family with identity link was used. Auto-regressive (AR1) correlation structure was selected since this was a time series (panel) data; we expected the correlation to decay as the outcome values were farther away from the time of interest.

**Covariates included in the model were age, sex, ethnicity, asthma/COPD, ARDS grade (none, mild, moderate or severe) at admission, history of CVD (diabetes, hypertension, coronary heart diseases or congestive heart failure), , and the baseline lowest FiO2. Other covariates included the use of any anticoagulation, therapeutic anticoagulation, hydroxychloroquine, azithromycin, and antivirals, (including remdesivir); vasopressors; and neuromuscular blockers**. The effect of adding steroids, IL-6 antagonists was assessed by including each variables as a dummy coded variable in the marginal model. In addition, an interaction term was added to the model to assess whether the combination of both drugs was associated with a statistically significant effect size.

inverse probability weighting (IPTW) are weights assigned to each observation across time conditioned on the previous exposure history, which are then multiplied to generate a single weight for a subject (4). Similar to conventional propensity score estimation, IPTW is generated using either a logit or probit model that regresses covariates to a treatment group (exposure) variable. With IPTW, the previous exposure history is incorporated to the propensity score estimation, which is time-varying. Standardized weights in a longitudinal setting are estimated. The numerator contains the probability of the observed exposure at each time point conditioned on the observed exposure history of the previous time point and the observed non-time varying covariates. The denominator contains the probability of the observed exposure at each time point conditioned on the observed exposure history of the previous time point, the observed time-varying covariates history at the current time point, and the non-time varying covariates. In standardized weights, the time-varying confounders are captured in the denominator but not in the numerator. However, the non-time varying (also known as fixed-time) covariates are captured in both the numerator. Due to the longitudinal nature of the data, the GLMMadaptive package was used for model fitting. The Package GLMMadaptive provides a suit of functions for fitting and post-processing mixed effects models for grouped/clustered outcomes which have a distribution other than a normal distribution. In particular, let yi denote a vector of grouped/clustered outcome for the i-th sample unit (i=1,…,n).

Standardized mean difference (SMD) were reported for comparison between cohorts before and after matching

**Handling of missing data:**

The generalized boosted model (GMB) creates a separate level during matching called NA and creates weights based on the distribution of missing values. So basically it considers NA as a level of its own. The weights are created for all respondents and are taking the missing values into consideration. Imputation requires that data is missing at random which is not the case here. Moreover, the variables we used had few missing values so there is no problem which would not have been the case if we used the SOFA and APACHE which had a lot of missing data.

**The pseudo population**

The populations are weighed by the probability of receiving treatments based on other confounders. The weights were produced using generalized boosted models and these weights were used when doing further analysis. The pseudo population is the sum of weights for each group rather than considering each observation as 1. Thus, the numbers can increase or decrease compared to the initial number of the group depends on the assigned weights. The aim to emulate randomized trial to create a pseudo study cohort, where the weighted version can balance off the covariate bias and mimic a randomized treatment assignment situation

**References :**

(1) Robins JM, Hernán MA, Brumback B. Marginal structural models and causal inference in epidemiology. Epidemiology. 2000;11:550-60.

(2) Heagerty PJ, Zeger SL. Marginalized multilevel models and likelihood inference (with comments and a rejoinder by the authors). Statist. Sci. 15(1): 1-26

(3) Hedeker, D., du Toit, S. H., Demirtas, H. and Gibbons, R. D. (2018), A note on marginalization of regression parameters from mixed models of binary outcomes. Biometrics 74, 354--361.

(4) Almirall D, Griffin BA, McCaffrey DF, Ramchand R, Yuen RA, Murphy SA. Time-varying effect moderation using the structural nested mean model: estimation using inverse-weighted regression with residuals. Stat Med. 2014 Sep 10;33(20):3466-87. doi: 10.1002/sim.5892. Epub 2013 Jul 19. PMID: 23873437; PMCID: PMC4008726.
