# Supplementary material for: Clinical characteristics and outcomes of critically ill mechanically ventilated COVID-19 patients receiving interleukin-6 receptor antagonists and corticosteroid therapy: a preliminary report from a multinational registry
Source: Eur J Med Res. 2021 Oct 2;26:117. doi: 10.1186/s40001-021-00591-x (PMC8487342; doi:10.1186/s40001-021-00591-x)
Supplement: Supplementary file 3 — Additional file 3. Labs and biomarkers data on day 7, 14, 21. [file 40001_2021_591_MOESM3_ESM.docx]

Table 1. Day 7 data

|  | **Steroids** | **IL-6 antagonists** | **Both** | **P** |
| --- | --- | --- | --- | --- |
|  | ***N=380*** | ***N=112*** | ***N=75*** |  |
| Leukocyte Count (highest) | 13.5 (6.49) | 14.0 (10.4) | 14.5 (9.73) | 0.870 |
| Leukocyte Count (Lowest) | 13.6 (6.68) | 14.4 (11.1) | 11.4 (5.40) | 0.539 |
| HCQ | 19 (5.00%) | 9 (8.04%) | 5 (6.67%) | 0.381 |
| Azithromycin: | 12 (3.16%) | 5 (4.46%) | 4 (5.33%) | 0.497 |
| Antiviral: | 50 (13.2%) | 16 (14.3%) | 9 (12.0%) | 0.901 |
| ARDS: |  |  |  | . |
| None | 241 (66.0%) | 91 (82.7%) | 49 (68.1%) |  |
| Mild (P:F 200-300) | 12 (3.29%) | 3 (2.73%) | 5 (6.94%) |  |
| Moderate (P:F 100-199) | 42 (11.5%) | 4 (3.64%) | 6 (8.33%) |  |
| Severe (P:F< 100) | 70 (19.2%) | 12 (10.9%) | 12 (16.7%) |  |
| Therapeutic anticoagulation: | 112 (29.5%) | 25 (22.3%) | 26 (34.7%) | 0.162 |
| Prophylactic anticoagulation: | 364 (95.8%) | 110 (98.2%) | 74 (98.7%) | 0.371 |
| pfo2 | 135 (71.8) | 174 (88.0) | 141 (85.3) | 0.093 |
| Sofa total | 7.12 (3.76) | 5.54 (3.54) | 6.69 (4.63) | 0.150 |
| Blood Glucose (highest) | 10.5 (4.27) | 9.84 (4.59) | 10.7 (3.52) | 0.724 |
| fio2_lowest | 0.51 (0.18) | 0.54 (0.16) | 0.59 (0.23) | 0.163 |
| fio2_highest | 0.66 (0.23) | 0.67 (0.24) | 0.67 (0.25) | 0.973 |
| fio2_time_gas | 0.68 (0.87) | 0.61 (0.23) | 0.61 (0.21) | 0.899 |
| Arterial PO2 | 85.5 (32.5) | 96.8 (30.2) | 87.2 (32.2) | 0.298 |
| PFio2 | 135 (71.8) | 174 (88.0) | 141 (85.3) | 0.093 |
| Platelets (Thrombocytes) (Lowest within 24 hours) | 278 (127) | 278 (102) | 261 (190) | 0.902 |
| Alanine Aminotransferase (ALT/SGPT) (highest) | 1.00 (0.91) | 1.14 (0.81) | 1.02 (0.49) | 0.699 |
| Aspartate aminotransferase (AST/ SGOT) (highest) | 1.01 (0.82) | 1.33 (1.04) | 0.91 (0.25) | 0.118 |
| Alkaline Phosphatase (Highest within 24 hours) | 1.74 (1.07) | 1.95 (1.36) | 2.49 (1.90) | 0.261 |
| Total bilirubin (highest) | 16.9 (14.5) | 12.5 (9.30) | 17.4 (16.6) | 0.248 |
| C-reactive protein (CRP) (Highest within 24 hours) | 122 (117) | 67.5 (78.0) | 77.9 (158) | 0.083 |
| Ferritin (Highest within 24 hours) | 1955 (2080) | 1091 (956) | 1434 (596) | 0.115 |
| Lactate Dehydrogenase (LDH) levels (Highest within 24 hours) | 10.7 (7.48) | 9.18 (4.19) | 8.15 (2.81) | 0.426 |
| Leukocyte Count (Lowest) | 13.6 (6.68) | 14.4 (11.1) | 11.4 (5.40) | 0.539 |
| Leukocyte Count (highest) | 13.5 (6.49) | 14.0 (10.4) | 14.5 (9.73) | 0.870 |
| Lymphocyte Count (Lowest within 24 hours) | 10.7 (12.5) | 15.8 (17.0) | 9.64 (5.45) | 0.135 |
| D-Dimer (Highest within 24 hours) | 2481 (3625) | 3116 (3072) | 13.3 (14.9) | 0.220 |
| Procalcitonin (Highest within 24 hours) | 2.96 (6.33) | 1.04 (2.58) | 0.89 (1.74) | 0.425 |
| Fibrinogen Level (Highest within 24 hours) | 5.43 (2.60) | 5.27 (2.52) | 3.44 (1.39) | 0.196 |
| Lactate (highest) | 2.14 (1.76) | 2.09 (0.92) | 2.14 (0.72) | 0.992 |
| Data was summarized using mean ± SD for continuous variables and counts (%) for categorical variables  Analysis was performed using Chi-square test of independence for categorical variables and one-way ANOVA for continuous variables | | | | |

Table 2. Day 14 data

|  | **Steroids** | **IL-6 antagonists** | **Both** | **P** |
| --- | --- | --- | --- | --- |
|  | ***N=207*** | ***N=59*** | ***N=49*** |  |
| Leukocyte Count (highest) | 13.5 (5.64) | 17.8 (19.3) | 9.55 (5.70) | 0.151 |
| Leukocyte Count (Lowest) | 13.2 (5.32) | 17.1 (20.0) | 9.50 (6.13) | 0.203 |
| HCQ | 5 (2.42%) | 1 (1.69%) | 4 (8.16%) | 0.095 |
| Azithromycin: | 2 (0.97%) | 0 (0.00%) | 1 (2.04%) | 0.474 |
| Antiviral: | 6 (2.90%) | 5 (8.47%) | 3 (6.12%) | 0.122 |
| ARDS: |  |  |  | 0.423 |
| None | 127 (64.8%) | 45 (78.9%) | 30 (65.2%) |  |
| Mild (P:F 200-300) | 8 (4.08%) | 3 (5.26%) | 3 (6.52%) |  |
| Moderate (P:F 100-199) | 24 (12.2%) | 3 (5.26%) | 5 (10.9%) |  |
| Severe (P:F< 100) | 37 (18.9%) | 6 (10.5%) | 8 (17.4%) |  |
| Therapeutic anticoagulation: | 54 (26.1%) | 17 (28.8%) | 16 (32.7%) | 0.636 |
| Prophylactic anticoagulation: | 190 (91.8%) | 58 (98.3%) | 48 (98.0%) | 0.1 |
| pfo2 | 130 (62.0) | 178 (122) | 141 (55.4) | 0.304 |
| Sofa total | 7.61 (3.84) | 6.77 (3.59) | 6.12 (3.40) | 0.491 |
| Blood Glucose (highest) | 9.93 (3.54) | 9.56 (3.83) | 7.36 (1.33) | 0.466 |
| fio2_lowest | 0.50 (0.18) | 0.43 (0.09) | 0.52 (0.26) | 0.340 |
| fio2_highest | 0.61 (0.24) | 0.56 (0.23) | 0.53 (0.25) | 0.633 |
| fio2_time_gas | 0.60 (0.22) | 0.51 (0.13) | 0.56 (0.26) | 0.458 |
| Arterial PO2 | 80.0 (34.2) | 79.0 (22.6) | 73.2 (17.4) | 0.901 |
| PFio2 | 130 (62.0) | 178 (122) | 141 (55.4) | 0.304 |
| Platelets (Thrombocytes) (Lowest within 24 hours) | 275 (142) | 262 (97.5) | 354 (273) | 0.393 |
| Alanine Aminotransferase (ALT/SGPT) (highest) | 0.79 (0.53) | 0.88 (0.60) | 0.77 (0.73) | 0.869 |
| Aspartate aminotransferase (AST/ SGOT) (highest) | 0.70 (0.37) | 0.93 (0.70) | 0.54 (0.31) | 0.179 |
| Alkaline Phosphatase (Highest within 24 hours) | 1.71 (0.77) | 1.97 (1.34) | 2.14 (1.36) | 0.583 |
| Total bilirubin (highest) | 12.9 (9.24) | 11.3 (8.85) | 8.13 (3.01) | 0.700 |
| C-reactive protein (CRP) (Highest within 24 hours) | 113 (121) | 49.9 (57.9) | 60.4 (76.6) | 0.268 |
| Ferritin (Highest within 24 hours) | 1036 (905) | 1034 (737) | 946 (1009) | 0.987 |
| Lactate Dehydrogenase (LDH) levels (Highest within 24 hours) | 7.33 (5.98) | 14.3 (24.8) | 6.25 (2.77) | 0.478 |
| Leukocyte Count (Lowest) | 13.2 (5.32) | 17.1 (20.0) | 9.50 (6.13) | 0.203 |
| Leukocyte Count (highest) | 13.5 (5.64) | 17.8 (19.3) | 9.55 (5.70) | 0.151 |
| Lymphocyte Count (Lowest within 24 hours) | 10.0 (7.27) | 26.3 (22.2) | 12.5 (7.58) | <0.001 |
| D-Dimer (Highest within 24 hours) | 3027 (2988) | 2152 (1957) | 349 (601) | 0.257 |
| Procalcitonin (Highest within 24 hours) | 4.30 (7.30) | 3.02 (4.04) | 0.21 (0.21) | 0.738 |
| Fibrinogen Level (Highest within 24 hours) | 5.07 (1.99) | 5.06 (1.69) | 3.40 (0.56) | 0.482 |
| Lactate (highest) | 1.50 (1.65) | 2.15 (1.50) | 1.88 (0.59) | 0.643 |
| Data was summarized using mean ± SD for continuous variables and counts (%) for categorical variables  Analysis was performed using Chi-square test of independence for categorical variables and one-way ANOVA for continuous variables | | | | |

Table 3. Day 21 data

|  | **Steroids** | **IL-6 antagonists** | **Both** | **P** |
| --- | --- | --- | --- | --- |
|  | ***N=60*** | ***N=22*** | ***N=27*** |  |
| Leukocyte Count (highest) | 12.0 (5.04) | 12.8 (0.60) | 13.2 (5.07) | 0.780 |
| Leukocyte Count (Lowest) | 11.9 (6.04) | 12.8 (0.60) | 11.8 (5.11) | 0.959 |
| HCQ | 0 (0.00%) | 0 (0.00%) | 1 (3.70%) | 0.45 |
| Azithromycin: | 0 (0.00%) | 0 (0.00%) | 1 (3.70%) | 0.45 |
| Antiviral: | 0 (0.00%) | 1 (4.55%) | 0 (0.00%) | 0.202 |
| ARDS: |  |  |  | 0.77 |
| None | 32 (55.2%) | 16 (72.7%) | 16 (64.0%) |  |
| Mild (P:F 200-300) | 3 (5.17%) | 1 (4.55%) | 2 (8.00%) |  |
| Moderate (P:F 100-199) | 10 (17.2%) | 1 (4.55%) | 3 (12.0%) |  |
| Severe (P:F< 100) | 13 (22.4%) | 4 (18.2%) | 4 (16.0%) |  |
| Therapeutic anticoagulation: | 8 (13.3%) | 5 (22.7%) | 6 (22.2%) | 0.409 |
| Prophylactic anticoagulation: | 53 (88.3%) | 21 (95.5%) | 24 (88.9%) | 0.76 |
| pfo2 | 166 (90.6) | 149 (101) | 132 (61.6) | 0.527 |
| Sofa total | 6.33 (4.32) | 6.10 (4.15) | 7.06 (3.43) | 0.797 |
| Blood Glucose (highest) | 9.06 (2.96) | 8.90 (4.84) | 11.7 (4.39) | 0.078 |
| fio2_lowest | 0.47 (0.20) | 0.47 (0.25) | 0.48 (0.25) | 0.982 |
| fio2_highest | 0.59 (0.27) | 0.54 (0.32) | 0.55 (0.27) | 0.827 |
| fio2_time_gas | 0.56 (0.25) | 0.47 (0.25) | 0.49 (0.31) | 0.654 |
| Arterial PO2 | 83.9 (26.6) | 162 (108) | 71.8 (27.5) | 0.001 |
| PFio2 | 166 (90.6) | 149 (101) | 132 (61.6) | 0.527 |
| Platelets (Thrombocytes) (Lowest within 24 hours) | 244 (110) | 243 (131) | 182 (108) | 0.234 |
| Alanine Aminotransferase (ALT/SGPT) (highest) | 0.72 (0.55) | 0.66 (0.36) | 1.10 (0.78) | 0.260 |
| Aspartate aminotransferase (AST/ SGOT) (highest) | 1.41 (2.27) | 0.57 (0.23) | 1.16 (0.82) | 0.688 |
| Alkaline Phosphatase (Highest within 24 hours) | 2.11 (1.13) | 1.64 (0.48) | 1.63 (0.49) | 0.437 |
| Total bilirubin (highest) | 9.20 (8.15) | 17.1 (13.8) | 17.0 (18.2) | 0.240 |
| C-reactive protein (CRP) (Highest within 24 hours) | 144 (99.2) | 54.8 (84.1) | 98.2 (125) | 0.449 |
| Ferritin (Highest within 24 hours) | 599 (806) | 490 (424) | 607 (681) | 0.968 |
| Lactate Dehydrogenase (LDH) levels (Highest within 24 hours) | 7.22 (2.85) | 5.78 (0.67) | 8.03 (3.98) | 0.667 |
| Leukocyte Count (Lowest) | 11.9 (6.04) | 12.8 (0.60) | 11.8 (5.11) | 0.959 |
| Leukocyte Count (highest) | 12.0 (5.04) | 12.8 (0.60) | 13.2 (5.07) | 0.780 |
| Lymphocyte Count (Lowest within 24 hours) | 12.7 (12.9) | 49.0 (55.1) | 8.89 (5.13) | 0.004 |
| D-Dimer (Highest within 24 hours) | 954 (1812) | 366 (489) | 34.2 (73.2) | 0.318 |
| Procalcitonin (Highest within 24 hours) | 0.62 (0.67) | 0.12 (0.04) | 0.62 (0.68) | 0.607 |
| Fibrinogen Level (Highest within 24 hours) | 4.98 (2.19) | 2.75 (1.95) | 4.29 (1.43) | 0.336 |
| Lactate (highest) | 2.01 (3.19) | 1.50 (1.41) | 1.85 (1.76) | 0.971 |
| Data was summarized using mean ± SD for continuous variables and counts (%) for categorical variables  Analysis was performed using Chi-square test of independence for categorical variables and one-way ANOVA for continuous variables | | | | |
