# Supplementary material for: Clinical characteristics and outcomes of critically ill mechanically ventilated COVID-19 patients receiving interleukin-6 receptor antagonists and corticosteroid therapy: a preliminary report from a multinational registry
Source: Eur J Med Res. 2021 Oct 2;26:117. doi: 10.1186/s40001-021-00591-x (PMC8487342; doi:10.1186/s40001-021-00591-x)
Supplement: Supplementary file 4 — Additional file 4. Collaborative authorship list. [file 40001_2021_591_MOESM4_ESM.docx]

**Collaborative Co-Author List.**

¥ On behalf of the Society of Critical Care Medicine Discovery Viral Infection and Respiratory Illness Universal Study (VIRUS): COVID-19 Registry Investigator Group

| **Belgium** |  |  |
| --- | --- | --- |
| Centre Hospitalier Jolimont: Jean-Baptiste Mesland, Pierre Henin, Hélène Petre, Isabelle Buelens, Anne-Catherine Gerard | | |
| The Brugmann University hospital, Bruxelles: Philippe Clevenbergh | | |
|  |  |  |
| **Bosnia and Herzegovina** | |  |
| University Clinical Hospital, Mostar, Bosnia and Herzegovina: Dragana Markotić, Ivana Bošnjak | | |
|  |  |  |
| **Columbia** |  |  |
| Clinica Medical SAS: Oscar Y Gavidia, Felipe Pachon, Yeimy A Sanchez | | |
|  |  |  |
| **Croatia** |  |  |
| Clinical Hospital Center Rijeka, Rijeka, Croatia: Danijel knežević | | |
|  |  |  |
| **Egypt** |  |  |
| Helwan University: Mohamed El Kassas, Mohamed Badr, Ahmed Tawheed, Ahmed Tawheed, Hend Yahia | | |
|  |  |  |
| **Hondurus** |  |  |
| Honduras Medical Center: Jose Luis Ramos Coello, Guillermo Perez, Ana Karen Vallecillo Lizardo, Gabina María Reyes Guillen, Helin Archaga Soto | | |
|  |  |  |
| **Hungary** |  |  |
| Uzsoki Teaching Hospital: Csaba Kopitkó, Ágnes Bencze,István Méhész, Zsófia Gerendai, | | |
|  |  |  |
| **India** |  |  |
| Jawaharlal Institute of Postgraduate Medical Education and Research, Pondicherry: Anusha Cherian, Sreejith Parameswaran , Magesh Parthiban, Menu Priya A. | | |
| Maulana Azad Medical College and Lok Nayak hospital: Mradul Kumar Daga, Munisha Agarwal , Ishan Rohtagi | | |
|  |  |  |
| **Japan** |  |  |
| Center Hospital of the National Center for Global Health and Medicine: Wataru Matsuda, Reina Suzuki | | |
| Sapporo City General Hospital: Yuki Itagaki, Akira Kodate, Reina Suzuki, Akira Kodate,Yuki Takahashi, Koyo Moriki | | |
| Tokyo Medical and Dental University: Hidenobu Shigemitsu, Yuka Mishima, Nobuyuki Nosaka, Michio Nagashima | | |
| Hiroshima University: Michihito Kyo | | |
|  |  |  |
| **Mexico** |  |  |
| Hospital Universitario, Universidad Autonoma de Nuevo León: Rene Rodriguez-Gutierrez, Jose Gerardo Gonzalez-Gonzalez, Alejandro Salcido-Montenegro, Adrian Camacho-Ortiz | | |
|  |  |  |
| **Pakistan** |  |  |
| Dow University Hospital: Muhammad Sohaib Asghar, Mashaal Syed, Syed Anosh Ali Naqvi | | |
|  |  |  |
| **Russia** |  |  |
| Kuban State Medical University with affiliation Territorial Hospital #2: Igor Borisovich Zabolotskikh, Konstantin Dmitrievich Zybin, Sergey Vasilevich Sinkov, Tatiana Sergeevna Musaeva | | |
|  |  |  |
| **Saudi Arabia** |  |  |
| King Saud University: Mohammed A Almazyad, Mohammed I Alarifi, Jara M Macarambon, Ahmad Abdullah Bukhari, Hussain A. Albahrani, Kazi N Asfina, Kaltham M Aldossary  King Saud bin Abdulaziz University for Health Sciences and King Abdullah International Medical Research Center: Yaseen M Arabi, Sheryl Ann Abdukahil | | |
| King Fahad Armed Forces Hospital: Razan K Alamoudi, Hassan M. AlSharif, Sarah A. Almazwaghi, Mohammed S Elsakran, Mohamed A Aid, Mouaz A Darwich, Omnia M Hagag, Salah A Ali, Alona rocacorba, Kathrine Supeña, Efren Ray Juane, Jenalyn Medina, Jowany Baduria | | |
| King Faisal Specialist Hospital & Research Centre - Riyadh: Marwa Ridha Amer, Mohammed Abdullah Bawazeer, Khalid Maghrabi, Abid Shahzad Butt, Talal I. Dahhan, Eiad Kseibi, Syed Moazzum Khurshid, Muath Rabee, Mohammed Abujazar, Razan Alghunaim, Maal Abualkhair, Abeer Turki AlFirm, Ali Al-Janoubi | | |
|  |  |  |
| **Serbia** |  |  |
| UMC Zvezdara, Belgrad: Bojan Kovacevic, Jovana Bojicic | | |
| Institute for Pulmonary Diseases of Vojvodina, Sremska Kamenica: Ana Andrijevic, Srdjan Gavrilovic, Vladimir Carapic | | |
|  |  |  |
| **Spain** |  |  |
| Hospital Universitario La Paz: Santiago Y. Teruel, Belen C. Martin, Santiago Y. Teruel | | |
|  |  |  |
| **United States** |  |  |
| Boston University School of Medicine, Boston, MA: Allan J. Walkey, Sushrut S. Waikar, Michael A. Garcia, Mia Colona, Zoe Kibbelaar, Michael Leong, Daniel Wallman, Kanupriya Soni, Jennifer Maccarone, Joshua Gilman, Ycar Devis, Joseph Chung, Munizay Paracha, David N. Lumelsky, Madeline DiLorenzo, Najla Abdurrahman, Shelsey Johnson | | |
| Albany Medical Center: Suzanne Barry, Christopher Woll, Gregory Wu, Erin Carrole, Kathryn Burke, Mustafa Mohammed | | |
| AnMed Health: Abhijit A Raval, Andrea Franks | | |
| Ascension St.Vincent Hospital: Anmol Kharbanda, Sunil Jhajhria, Zachary Fyffe | | |
| Ascension/St. Thomas Research Institute West Campus: Stephen Capizzi, Bethany Alicie, Martha Green, Lori Crockarell, Amelia Drennan, Kathleen Dubuque, Tonya Fambrough, Nikole Gasaway, Briana Krantz, Peiman Nebi, Jan Orga, Margaret Serfass, Alina Simion, Kimberly Warren, Cassie Wheeler, CJ Woolman | | |
| Augusta Health: Andrew S. Moyer, George M. Verghese | | |
| Augusta University Medical Center: Andrea Sikora Newsome, Christy C. Forehand, Rebecca Bruning, Timothy W. Jones | | |
| Banner University Medical Center-Tucson: Jarrod M Mosier, Karen Lutrick, Beth Salvagio Campbell, Cathleen Wilson, Patrick Rivers, Jonathan Brinks, Mokenge Ndiva Mongoh, Boris Gilson | | |
| Baylor College of Medicine, Baylor St. Lukes Medical Center: Christopher M Howard, Cameron McBride, Jocelyn Abraham, Orlando Garner, Katherine Richards, Keegan Collins, Preethi Antony, Sindhu Mathew | | |
| Baylor Scott & White Health: Valerie C. Danesh, Gueorgui Dubrocq, Amber L. Davis, Marissa J Hammers, ill M. McGahey, Amanda C. Farris, Elisa Priest, Robyn Korsmo, Lorie Fares, Kathy Skiles, Susan M. Shor, Kenya Burns, Corrie A Dowell, Gabriela “Hope” Gonzales, Melody Flores, Lindsay Newman, Debora A Wilk, Jason Ettlinger, Jaccallene Bomar, Himani Darji, Alejandro Arroliga, Alejandro C Arroliga, Corrie A. Dowell, Gabriela Hope Conzales, Melody Flores, Lindsay Newman, Debora A. Wilk, Jason Ettlinger, Himani Darji, Jaccallene Bomar | | |
| Beth Israel Deaconess Medical Center: Valerie M. Banner-Goodspeed, Somnath Bose, Lauren E. Kelly, Melisa Joseph, Marie McGourty, Krystal Capers, Benjamin Hoenig, Maria C. Karamourtopoulos, Anica C. Law, Elias N. Baedorf Kassis | | |
| Cedars Sinai Medical Center: Pooja A. Nawathe, Isabel Pedraza, Jennifer Tsing, Karen Carr, Anila Chaudhary, Kathleen Guglielmino | | |
| Chambersburg Hospital: Raghavendra Tirupathi, Alymer Tang, Arshad Safi, Cindy Green, Jackie Newell | | |
| Children's Hospital Colorado, University of Colorado Anschutz Medical Campus: Katja M. Gist, Imran A Sayed, John Brinton, Larisa Strom | | |
| Children's Hospital of Philadelphia: Kathleen Chiotos, Allison M. Blatz, Giyoung Lee, Ryan H. Burnett, Guy I. Sydney, Danielle M. Traynor | | |
| Cox Medical Center Springfield: Steven K. Daugherty, Sam Atkinson, Kelly Shrimpton | | |
| Detar Family Medicine residency: Sidney Ontai, Brian Contreras, MD, Uzoma Obinwanko, Nneka Amamasi, Amir Sharafi | | |
| Detroit Medical Centre: Sarah Lee, Zahia Esber, Chetna Jinjvadia | | |
| George Washington University: David P. Yamane, Ivy Benjenk, Nivedita Prasanna | | |
| Howard University Hospital: Orma Smalls | | |
| Jacobs Medical Center UC San Diego Health – La Jolla: Atul Malhotra, Abdurrahman Husain, Qais Zawaydeh | | |
| JPS Health Network: Steven Q. Davis, Valentina Jovic, Valentina Jovic, Max Masuda, Amanda Hayes | | |
| Lakes Region General Hospital: Michael Smith, William Snow, Riley Liptak, Hannah Durant, Valerie Pendleton, Alay Nanavati, Risa Mrozowsk | | |
| LifeBridge Health/Sinai and Northwest Hospitals: Namrata Nag, Jeff Brauer, Ashwin Dharmadhikari, Sahib Singh, Franco Laghi,  Ghania Naeem, Andrew Wang, Kevin Bliden, Amit Rout, Jaime Barnes, Martin Gesheff, Asha Thomas, Melbin Thomas, Alicia R. Liendo, Jovan Milosavljevic, Kenan Abbasi, Nicholas B. Burley, Nicole Rapista, Samuel Amankwah, Sanjay K Poudel, Saroj  Timilsina, Sauradeep Sarkar, Oluwasayo Akinyosoye, Shashi K. Yalamanchili, Sheena Moorthy, Sonia Sugumar, Jonathan Ford, Martin C. Taylor, Charlotte Dunderdale, Alyssa Henshaw, Mary K. Brunk, Jessica Hagy,  Shehryar Masood, Sushrutha Sridhar | | |
| Loyola University Medical Center: Yuk Ming Liu, Sarah Zavala, Sarah Zavala, Esther Shim | | |
| M Health-Fairview, University of Minnesota: Ronald A. Reilkoff, Julia A. Heneghan, Sarah Eichen, Lexie Goertzen, Scott Rajala, Ghislaine Feussom, Ben Tang | | |
| MacNeal Hospital Loyola Medicine: Christine C. Junia, Robert Lichtenberg, Hasrat Sidhu, Diana Espinoza, Shelden Rodrigues, Maria Jose Zabala, Daniela Goyes, Ammu Susheela, Buddhi Hatharaliyadda, Naveen Rameshkumar, Amulya Kasireddy, Genessis Maldonado, Lisseth Beltran, Akshata Chaugule, Hassan Khan | | |
| Mayo Clinic Arizona: Rodrigo Cartin-Ceba, Ayan Sen, Amanda Palacios, Giyth M. Mahdi | | |
| Mayo Clinic Rochester: Rahul Kashyap, Ognjen Gajic, Vikas Bansal, Aysun Tekin, Amos Lal, John C. O'Horo, Neha N. Deo, Mayank Sharma, Shahraz Qamar, Cory J. Kudrna , Juan Pablo Domecq | | |
| Mayo Clinic, Eau Claire: Abigail T. La Nou, Marija Bogojevic | | |
| Mayo Clinic, Florida: Devang Sanghavi, Pramod Guru, Karthik Gnanapandithan, Hollie Saunders, Zachary Fleissner, Juan Garcia , Alejandra Yu Lee Mateus, Siva Naga Yarrarapu | | |
| Mayo Clinic, Mankato: Syed Anjum Khan, Nitesh Kumar Jain, Thoyaja Koritala | | |
| Medical Center Health System, Odessa: Alexander Bastidas, Gabriela Orellana, Adriana Briceno Bierwirth, Eliana Milazzo, Juan Guillermo Sierra, Thao Dang | | |
| Medical Center Navicent Health: Amy B. Christie, Dennis W. Ashley, Rajani Adiga | | |
| Mercy Hospital and Medical Center, Chicago: Travis Yamanaka, Nicholas A. Barreras, Michael Markos, Anita Fareeduddin, Rohan Mehta | | |
| Mercy Hospital, Saint Louis: Chakradhar Venkata, Miriam Engemann, Annamarie Mantese | | |
| Millard Fillmore Suburban Hospital: Anna Eschler, Mary Hejna, Emily Lewandowski, Kristen Kusmierski, Clare Martin | | |
| Montefiore Medical Center The Bronx: Jen-Ting Chen, Aluko Hope, Zoe Tsagaris, Elise Ruen, Aram Hambardzumyan | | |
| OSF Saint Francis Medical Center: Bhagat S. Aulakh, Sandeep Tripathi, Jennifer A. Bandy, Lisa M. Kreps, Dawn R. Bollinger, Jennifer A. Bandy | | |
| OSF Saint Francis Medical Center: Bhagat S. Aulakh, Sandeep Tripathi, Jennifer A. Bandy, Lisa M. Kreps, Dawn R. Bollinger, Jennifer A. Bandy | | |
| Parkview Health System, Fort Wayne: Roger Scott Stienecker, Andre G. Melendez, Tressa A. Brunner, Sue M Budzon, Jessica L. Heffernan, Janelle M. Souder, Tracy L. Miller, Andrea G. Maisonneuve | | |
| Samaritan Health Services: Brian L. Delmonaco, Anthony Franklin, Mitchell Heath | | |
| Sarasota Memorial Hospital: Antonia L. Vilella, Sara B. Kutner, Kacie Clark, Danielle Moore | | |
| St. Joseph's Candler Health System: Howard A. Zaren, Stephanie J. Smith, Grant C. Lewis, Lauren Seames, Cheryl Farlow, Judy Miller, Gloria Broadstreet | | |
| St.Mary Medical Center, Langhorne: Umang Patel, Jordesha Hodge, KrunalKumar Patel, Shivani Dalal, Himanshu Kavani, Sam Joseph | | |
| Stamford Health: Michael A. Bernstein, Ian K. Goff, Matthew Naftilan, Amal Mathew, Deborah Williams, Sue Murdock, RN, Maryanne Ducey, Kerianne Nelson | | |
| Stanford Hospital and Clinics: Paul K Mohabir, Connor G O'Brien, Komal Dasani | | |
| The Children's Hospital at OU Medicine: Neha Gupta, Tracy L Jones, Shonda C Ayers, Amy B Harrell, Dr. Brent R Brown | | |
| The University of Tennessee Medical Center: Megan Edwards, Caleb Darby, Kristy Page, Amanda Brown, Jessie McAbee | | |
| Thomas Jefferson University Hospital: Katherine A. Belden, Michael Baram, Devin M. Weber, Rosalie DePaola, Yuwei Xia, Hudson Carter, Aaron Tolley, Mary Barletta | | |
| Truman Medical Centers: Mark Steele, Laurie Kemble | | |
| Tulane University Medical Center and University Medical Center New Orleans: Joshua L. Denson, A. Scott Gillet, Margo Brown, Rachael Stevens, Andrew Wetherbie, Kevin Tea, Mathew Moore | | |
| UNC Medical Center: Benjamin J Sines, Thomas J Bice | | |
| University Medical Center of Southern Nevada Las Vegas; University of Nevada, Las Vegas: Rajany V. Dy, Alfredo Iardino, Jill Sharma, Julia Christopher, Marwan Mashina, Kushal Patel | | |
| University of Alabama at Birmingham: Erica C. Bjornstad, Nancy M. Tofil, Scott House, Isabella Aldana | | |
| University of Arkansas for Medical Sciences: Nikhil K. Meena, Jose D. Caceres, Nikhil K Meena, Sarenthia M. Epps, Harmeen Goraya, Kelsey R. Besett, MD, Ryan James, Lana Y. Abusalem, Akash K. Patel, Lana S Hasan | | |
| University of Cincinnati: Dina Gomaa B.S., Michael Goodman, Devin Wakefield, Anthony Spuzzillo, John O. Shinn II | | |
| University of Iowa Carver College of Medicine: Patrick W. McGonagill, Colette Galet, Janice Hubbard, David Wang, Lauren Allan, Aditya Badheka, Madhuradhar Chegondi | | |
| University of Kansas Medical Center: Usman Nazir, Garrett Rampon, Jake Riggle, Nathan Dismang | | |
| University of Louisville Hospital: Ozan Akca, Rainer Lenhardt, Rodrigo S. Cavallazzi, Ann Jerde, Alexa Black, Allison Polidori, Haily Griffey, Justin Winkler, Thomas Brenzel | | |
| University of Michigan Health System: Pauline Park, Andrew Admon, Sinan Hanna, Rishi Chanderraj, Maria Pliakas, Ann Wolski, Jennifer Cirino | | |
| University of Missouri, Columbia: Dima Dandachi, Hariharan Regunath, Maraya N. Camazine, Grant. E. Geiger, Abdoulie O. Njai, Baraa M. Saad | | |
| University of Vermont Larner College of Medicine: Renee D. Stapleton, Anne E. Dixon, Olivia Johnson, Sara S. Ardren, Stephanie Burns, Anna Raymond, Erika Gonyaw, Kevin Hodgdon, Chloe Housenger, Benjamin Lin, Karen McQuesten, Heidi Pecott-Grimm, Julie Sweet, Sebastian Ventrone | | |
| Wake Forest University School of Medicine; Wake Forest Baptist Health Network: Ashish K. Khanna, Lynne Harris, Bruce Cusson, Jacob Fowler, David Vaneenenaam, Glen McKinney, Imoh Udoh, Kathleen Johnson | | |
| Yale New Haven Health New Haven: Kevin Sheth, Abdalla Ammar, Mahmoud Ammar, Victor Torres Lopez , Charles Dela Cruz, Akhil Khosla, Samir Gautam | | |
|  |  |  |
